# Supplementary material for: Stoichiometry validation of supramolecular complexes with a hydrocarbon cage host by van ’t Hoff analyses
Source: Nat Commun. 2023 Dec 21;14:8246. doi: 10.1038/s41467-023-43979-5 (PMC10739680; doi:10.1038/s41467-023-43979-5)
Supplement: Supplementary file 1 — Supplementary Information [file 41467_2023_43979_MOESM1_ESM.pdf]

Supplementary Information

Stoichiometry validation of supramolecular complexes with a hydrocarbon cage host by van 't Hoff analyses

Toshiya M. Fukunaga, Yuzuka Onaka, Takahide Kato, Koki Ikemoto, Hiroyuki Isobe

Table of Contents

Supplementary Methods 1
General 1
Materials 1
Synthesis 2
Crystallography 4
Titration experiments and fitting analyses 6
Theoretical calculations 30
Spectra 31
Supplementary References 39

Supplementary Methods

General

Flash silica gel column chromatography was performed on silica gel 60N (spherical and neutral gel, 40-50 μm, Kanto). Gel permeation chromatography (GPC) was performed on JAI LC-5060 systems with JAI GEL 2HR-40 and 2.5HR-40 columns (eluent: chloroform) under UV detections. ^{1}H and ^{13}C NMR spectra were recorded on a JEOL RESONANCE JNM-ECA II 600 spectrometer (^{1}H: 600 MHz and ^{13}C: 151 MHz) equipped with an UltraCOOL probe. Chemical shift values are given with respect to internal $CHCl_3$ for ^{1}H NMR (δ 7.26) and $CDCl_3$ for ^{13}C NMR (δ 77.16). Methyl (CH_3), methylene (CH_2), and methine (CH) signals in ^{13}C NMR spectra were assigned by DEPT135 spectra. High-resolution mass spectra were recorded on a Bruker micrOTOF II (APCI, ionization mode: positive) or on a Bruker Daltonics autoflex speed (MALDI) with pyrene as a matrix (ionization mode: reflector positive). A precision balance (XPE205V, Mettler Toledo) was used for titration experiments.

Materials

All the reactions were performed under N_2 atmosphere by using anhydrous solvents purified by a solvent purification system (GlassContour) equipped with columns of activated alumina and supported copper catalyst (Q-5).[1] Compound **2** was prepared according to the reported procedures.[2]

All other chemicals purchased from TCI, Wako Pure Chemical Industries, Kanto Chemical, Sigma-Aldrich and BLDpharm were of reagent grade and were used without any further purification.

Synthesis

Compound 3

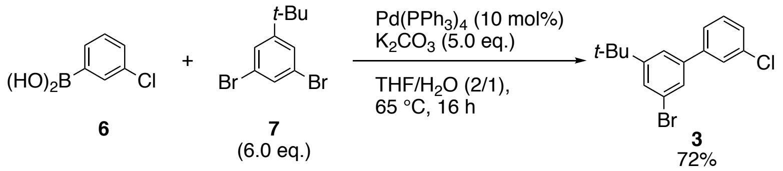

To a solution of 3-chlorobenzene boronic acid (**6**) (313 mg, 2.00 mmol), 1,3-dibromo-5-*t*-butylbenzene (**7**) (3.50 g, 12.0 mmol), and $Pd(PPh_3)_4$ (231 mg, 0.200 mmol) in THF (13 mL) were added 5 M aq. K_2CO_3 (6.6 mL, 10.0 mmol). The mixture was stirred at 65 °C for 16 h. After the addition of water (20 mL), the organic layer was separated, and the aqueous layer was extracted with ethyl acetate (10 mL × 2). The combined organic layer was washed with brine (30 mL), dried over Na_2SO_4, and concentrated in vacuo. A crude material was purified by silica gel column chromatography (eluent: hexane) to give the title compound in 72% yield (468 mg, 1.44 mmol). ^{1}H NMR (600 MHz, $CDCl_3$) δ 7.53-7.52 (m, 2H), 7.51 (t, J = 1.8 Hz, 1H), 7.46 (t, J = 1.8 Hz, 1H), 7.42 (dt, J = 7.2 Hz, 1.8 Hz , 1H), 7.37 (t, J = 7.2 Hz, 1H), 7.34 (dt, J = 7.2 Hz, 1.8 Hz , 1H), 1.36 (s, 9H); ^{13}C NMR (151 MHz, $CDCl_3$) δ 154.2, 142.3, 141.7, 134.9, 130.2 (CH), 128.2 (CH), 127.9 (CH), 127.5 (CH), 127.5 (CH), 125.6 (CH), 123.2 (CH), 123.0, 35.2, 31.4 (CH_3); HRMS (APCI) (*m*/*z*): $[M]^+$ calcd. for $C_{16}H_{16}BrCl$ 322.0118, found 322.0115.

Compound 4

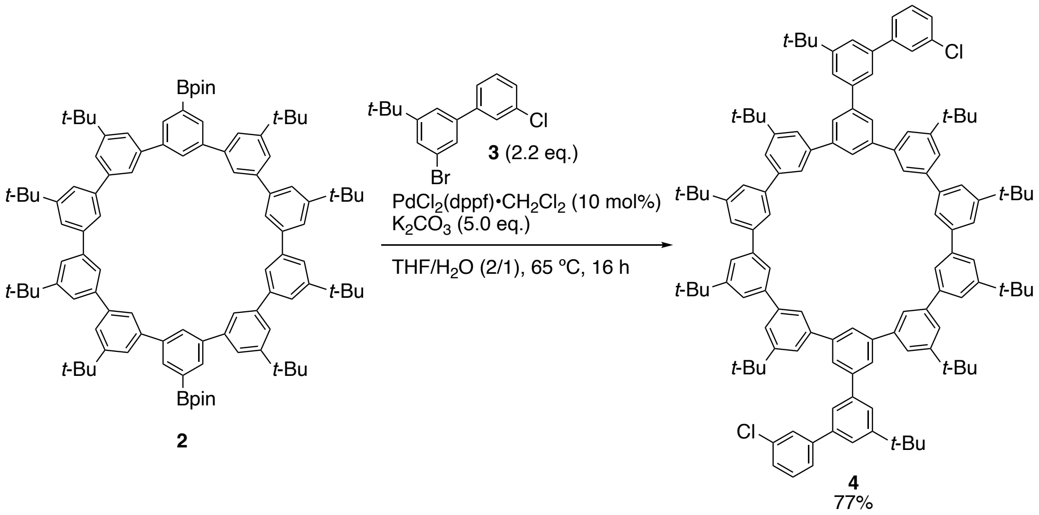

To a solution of **2** (73.1 mg, 50.0 μmol), **3** (35.6 mg, 110 μmol), and $PdCl_2(dppf)\cdot CH_2Cl_2$ (4.10 mg, 5.00 μmol) in THF (1.7 mL) were added 0.3 M aq. K_2CO_3 (0.83 mL, 25.0 μmol). The mixture was stirred at 65 °C for 16 h. After the addition of water (5 mL), the organic layer was separated, and the

aqueous layer was extracted with chloroform (5 mL × 3). The combined organic layer was washed with brine (15 mL), dried over Na_2SO_4, and concentrated in vacuo. A crude material was purified by GPC to give the title compound in 77% yield (65.1 mg, 38.4 μmol). ^{1}H NMR (600 MHz, $CDCl_3$) δ 7.80 (s, 4H), 7.77 (s, 2H), 7.70 (s, 2H), 7.66 (s, 2H), 7.65-7.62 (m, 14H), 7.61-7.58 (m, 14H), 7.53 (d, J = 7.8 Hz, 2H), 7.38 (t, J = 7.8 Hz, 2H), 7.34 (d, J = 7.8 Hz, 2H), 1.43 (s, 54H), 1.42 (s, 36H); ^{13}C NMR (151 MHz, $CDCl_3$) δ 152.7, 152.3, 152.1, 149.3, 143.6, 143.5, 142.8, 142.7, 142.3, 142.1, 141.9, 140.4, 134.8, 130.1 (CH), 127.6 (CH), 127.5 (CH), 126.3 (CH), 126.1 (CH), 125.7 (CH), 124.5 (CH), 124.5 (CH), 124.4 (CH), 124.3 (CH), 124.1 (CH), 124.0 (CH), 124.0 (2 overlapping CH), 123.7 (CH), 35.2, 35.2, 35.1, 31.6 (2 overlapping CH_3), 31.6 (CH_3); HRMS (MALDI-TOF) (m/z): $[M]^+$ calcd. for $C_{124}H_{134}Cl_2$ 1692.9857, found 1692.9879.

Phenine polluxene 1a

The purity of the final target was confirmed by high-performance liquid chromatography (HPLC) with COSMOSIL BuckyPrep (4.6ϕ × 250 mm) or COSMOSIL πNAP (4.6ϕ × 250 mm) (Supplementary Fig. 1).

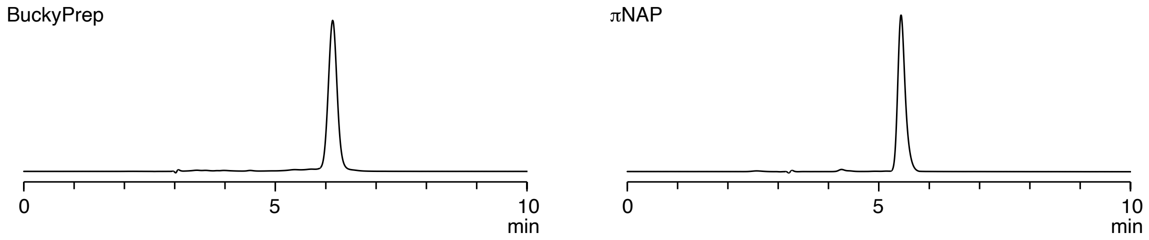

Supplementary Fig. 1 | Chromatograms of purified phenine polluxene 1a. Two different columns were used to assure the purity (COSMOSIL BuckyPrep, 4.6ϕ × 250 mm and COSMOSIL πNAP, 4.6ϕ × 250 mm). Chromatographic conditions: flow rate = 1.0 mL min^{-1}, eluent = 50% MeOH/$CHCl_3$, UV detection = 300 nm.

Phenine polluxene 1c

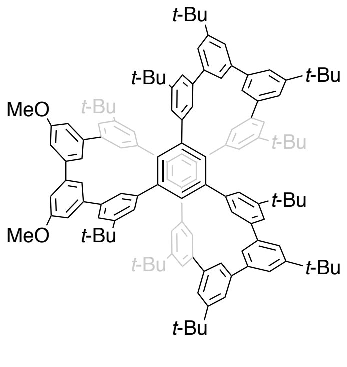

Phenine polluxene (**1c**) with methoxy substituents was synthesized by using similar methods and routes reported for **1a**. 84% yield (21.9 mg). ^{1}H NMR (600 MHz, $CDCl_3$) δ 7.93 (s, 2H), 7.91 (s, 4H), 7.78 (s, 6H), 7.77 (s, 4H), 7.74 (s, 4H), 7.64 (s, 6H), 7.59-7.54 (m, 14H), 7.13 (s, 2H), 3.98 (s, 6H), 1.50 (s, 36H), 1.38 (s, 36H), 1.37 (s, 18H); ^{13}C NMR (151 MHz, $CDCl_3$) δ 160.4, 152.4, 152.3, 152.2, 144.6, 143.1, 143.1, 142.9, 142.8, 142.3, 142.3, 141.3, 141.2, 141.1, 125.8 (CH), 125.8 (CH), 124.8 (CH), 124.5 (CH), 124.4 (CH), 124.3 (CH), 124.2 (CH), 123.8 (2 overlapping CH), 123.5 (CH), 123.1 (CH), 118.3 (CH), 113.4 (CH), 111.7 (CH), 55.7 (CH_3), 35.2, 35.1, 35.1, 31.6 (2 overlapping CH_3), 31.6 (CH_3); HRMS (MALDI-TOF) (m/z): $[M]^+$ calcd. for $C_{126}H_{138}O_2$ 1683.0961, found 1683.0636.

Crystallography

Detailed data for the diffraction analyses are given in Supplementary Table 1. Alert B from CheckCIF does not question conclusion of molecular structures.

Supplementary Table 1 | Crystal data of 1a

Data deposition	CCDC 2281309	
Empirical formula	$C_{63.17}H_{68.17}Cl_{3.51}$	
Formula weight	951.81	
Temperature	95(2) K	
Wavelength	0.9000 Å	
Crystal system	Monoclinic	
Space group	*C*2/*c*	
Unit cell dimensions	a = 28.640(6) Å	α = 90°
	b = 16.270(3) Å	β = 99.32(3)°
	c = 25.640(5) Å	γ = 90°
Volume	11790(4) Å^3	
Z	8	
Calculated density	1.072 Mg/m^3	
Absorption coefficient	0.401 mm^{-1}	
F(000)	4055	
Crystal size	0.200 × 0.020 × 0.020 mm^3	
Radiation	synchrotron (λ = 0.9000 Å)	
Theta range for data collection	1.829° to 32.785°	
Index ranges	$-34 \le h \le 34$, $-19 \le k \le 19$, $-27 \le l \le 27$	
Reflections collected	128239	
Independent reflections	10253 [R_{int} = 0.0990]	
Completeness to theta = 32.684	95.4 %	
Absorption correction	Semi-empirical from equivalents	
Max. and min. transmission	1.000 and 0.704	
Refinement method	Full-matrix least-squares on F^2	
Data / restraints / parameters	10253 / 743 / 743	
Goodness-of-fit on F^2	1.778	
Final R indices [I>2σ(I)]	R_1 = 0.1481, wR_2 = 0.4402	
R indices (all data)	R_1 = 0.1502, wR_2 = 0.4461	
Largest diff. peak and hole	1.607 / –1.247 e•Å^{-3}	

Crystal structures of **1a** (R = H; CCDC 2281309, this study) and **1b** (R = *t*-Bu; CCDC 211285, ref. 2) are compared in detail including solvent molecules (Supplementary Fig. 2a). For **1a**, residual electron densities were not fully assigned and were eliminated by SQUEEZE. The eliminated density is shown in Supplementary Fig. 2b.

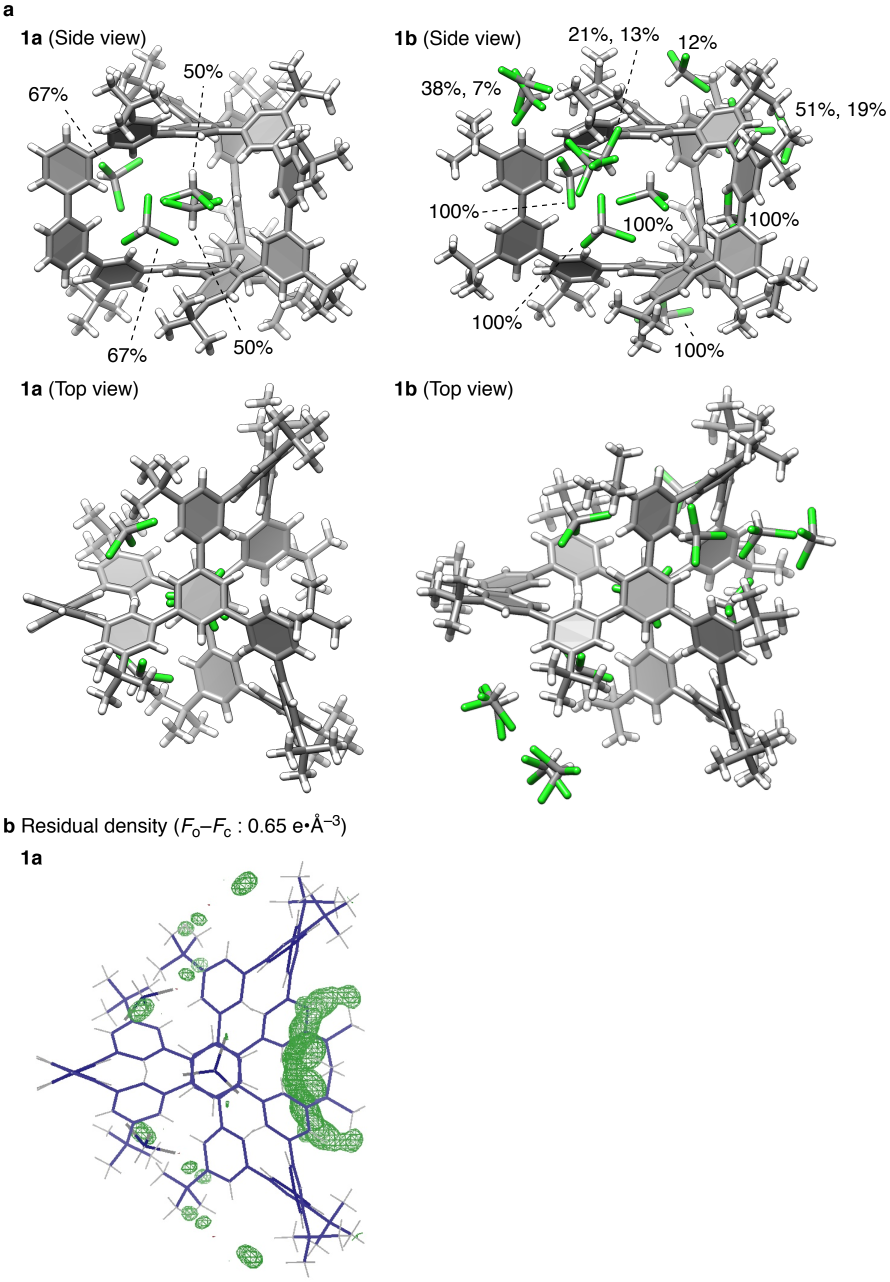

Supplementary Fig. 2 | Comparisons of crystal structures of 1a and 1b. (a) Molecular structures. Occupancies of chloroform molecules are shown. (b) Residual density eliminated from the crystal data of **1a** by SQUEEZE.

Titration experiments and fitting analyses

1 *⊃$(CHCl_3)_n$*

Procedures of fitting analyses are described. Initial concentrations of host and guest before the titration are defined as $[H]_i$ and $[G]_i$, respectively. Total concentrations of host and guest during the titration are defined as $[H]_0$ and $[G]_0$ (observables), respectively, and are described as a function of $[H]_i$ and $[G]_i$ in following equations,

$$[\mathrm{H}]_0 = \frac{[\mathrm{H}]_\mathrm{i}[\mathrm{G}]_\mathrm{i}}{[\mathrm{G}]_\mathrm{i} + [\mathrm{H}]_\mathrm{i}x} \quad (1),$$

$$[\mathrm{G}]_0 = [\mathrm{H}]_0 x \quad (2),$$

where $x = [G]_0/[H]_0$.

○1:1 complex, **1**⊃$(CHCl_3)_1$

Upon formation of 1:1 complex, the total concentration of host is defined as a sum of concentrations of host ([H]) and complex ([HG]) in solution, and the total concentration of guest is defined as a sum of concentrations of guest ([G]) and complex ([HG]) in solution.

$$[\mathrm{H}]_0 = [\mathrm{H}] + [\mathrm{HG}] \quad (3).$$

$$[\mathrm{G}]_0 = [\mathrm{G}] + [\mathrm{HG}] \quad (4).$$

For **1:1**, the association constant K_1 is then defined as

$$K_1 = \frac{[\mathrm{HG}]}{[\mathrm{H}][\mathrm{G}]} \quad (5).$$

By using (3)-(5), the association constant and the total concentrations are correlated in a quadratic equation

$$K_1[\mathrm{HG}]^2 - (K_1[\mathrm{G}]_0 + K_1[\mathrm{H}]_0 + 1)[\mathrm{HG}] + K_1[\mathrm{G}]_0[\mathrm{H}]_0 = 0 \quad (6),$$

and the concentration of 1:1 complex is thus described as

$$[\mathrm{HG}] = \frac{1}{2}\left\{\left([\mathrm{G}]_0 + [\mathrm{H}]_0 + \frac{1}{K_1}\right) - \sqrt{\left([\mathrm{G}]_0 + [\mathrm{H}]_0 + \frac{1}{K_1}\right)^2 - 4[\mathrm{H}]_0[\mathrm{G}]_0}\right\} \quad (7).$$

During the titration, we experimentally track the change of the chemical shift of guest ($\Delta\delta$), which obeys an equation

$$\Delta\delta = \delta_{\Delta\mathrm{HG}}\frac{[\mathrm{HG}]}{[\mathrm{G}]_0} \quad (8),$$

where $\delta_{\Delta HG}$ is a difference of chemical shift of host and 1:1 complex. By applying (7) to (8), we obtain

$$\Delta\delta = \frac{\delta_{\Delta\mathrm{HG}}}{2}\left\{\left(1 + \frac{[\mathrm{H}]_0}{[\mathrm{G}]_0} + \frac{1}{K_1[\mathrm{G}]_0}\right) - \sqrt{\left(1 + \frac{[\mathrm{H}]_0}{[\mathrm{G}]_0} + \frac{1}{K_1[\mathrm{G}]_0}\right)^2 - \frac{4[\mathrm{H}]_0}{[\mathrm{G}]_0}}\right\} \quad (9),$$

which is used for the fitting analyses.

Plotting the observed $\Delta\delta$ values on the y axis against $[G]_0/[H]_0$ in the x axis affords isotherms in the $[CHCl_3]_0/[\mathbf{1}]_0$-$\Delta\delta$ graphs (Supplementary Fig. 3), which were fitted by using (9). Specifically, using (1), (2) and (9), the codes to minimize "f" are written as follows:

```
double H0 = Hi*Gi/(Gi+Hi*x);
double G0 = H0*x;
f=y-0.5*D*((1+H0/G0+1/(K*G0))-sqrt((1+H0/G0+1/(K*G0))^2-4*H0/G0));
```

where `D` stands for $\delta_{\Delta HG}$ and `K` stands for K_1, and running the codes on OriginPro affords the association constant (K_1), the chemical shift ($\delta_{\Delta HG}$) and sum of squared residuals ($SS_{\mathbf{1:1}}$).

○1:2 complex, $\mathbf{1}\supset(CHCl_3)_2$

Upon formation of 1:2 complex, the total concentration of host is defined as a sum of concentrations of host ([H]), 1:1 complex ([HG]) and 1:2 complex ($[HG_2]$) in solution, and the total concentration of guest is defined as a sum of concentrations of guest ([G]), 1:1 complex ([HG]) and 1:2 complex ($[HG_2]$) in solution.

$$[\mathrm{H}]_0 = [\mathrm{H}] + [\mathrm{HG}] + [\mathrm{HG}_2] \tag{10}$$

$$[\mathrm{G}]_0 = [\mathrm{G}] + [\mathrm{HG}] + 2[\mathrm{HG}_2] \tag{11}$$

For **1:2**, the association constants K_1 and K_2 are then defined as

$$K_1 = \frac{[\mathrm{HG}]}{[\mathrm{H}][\mathrm{G}]} \tag{12}$$

$$K_2 = \frac{[\mathrm{HG}_2]}{[\mathrm{HG}][\mathrm{G}]} \tag{13}$$

By using (10)-(13), the association constant and the total concentrations are correlated in a cubic equation

$$K_1K_2[\mathrm{G}]^3 + (2K_1K_2[\mathrm{H}]_0 - K_1K_2[\mathrm{G}]_0 + K_1)[\mathrm{G}]^2 + (K_1[\mathrm{H}]_0 - K_1[\mathrm{G}]_0 + 1)[\mathrm{G}] - [\mathrm{G}]_0 = 0 \tag{14}$$

During the titration, we experimentally track the change of the chemical shift of guest ($\Delta\delta$), which obeys an equation

$$\Delta\delta = \delta_{\Delta\mathrm{HG}}\frac{[\mathrm{HG}]}{[\mathrm{G}]_0} + 2\delta_{\Delta\mathrm{HG}_2}\frac{[\mathrm{HG}_2]}{[\mathrm{G}]_0} \tag{15}$$

where $\delta_{\Delta HG}$ is a difference of chemical shift of host and 1:1 complex, and $\delta_{\Delta HG2}$ is a difference of chemical shift of host and 1:2 complex. By applying (10)-(13) to (15), we obtain

$$\Delta\delta = \frac{\delta_{\Delta\mathrm{HG}}K_1[\mathrm{G}] + 2\delta_{\Delta\mathrm{HG}_2}K_1K_2[\mathrm{G}]^2}{1 + K_1[\mathrm{G}] + K_1K_2[\mathrm{G}]^2}\cdot\frac{[\mathrm{H}]_0}{[\mathrm{G}]_0} \tag{16}$$

which is used for the fitting analyses.

Plotting the observed $\Delta\delta$ values on the y axis against $[G]_0/[H]_0$ in the x axis affords isotherms in the

$[CHCl_3]_0/[\mathbf{1}]_0$-$\Delta\delta$ graphs (Supplementary Fig. 3), which were fitted by using (16). Specifically, using (1), (2) and (10)-(16), the codes to minimize "`f`" are written as follows:

```
double H0 = Hi*Gi/(Gi+Hi*x);
double G0 = H0*x;
double K = K1*K2;
double L = K1+K1*K2*(2*H0-G0);
double M = K1*(H0-G0)+1;
double N = -G0;
double O = -L/(3*K);
double P = M/K-L*L/(3*K*K);
double Q = N/K-M*L/(3*K*K)+2*L*L/(27*K*K*K);
double R = Q*Q/4+P*P*P/27;
double S;
if(R>0)
{
       S = -Q/2+sqrt(R);
}
       else
{
       S = -Q/2;
}
double T;
if(R>0)
{
       T = 0;
}
else
{
       T = sqrt(-R);
}
double U = sqrt(S*S+T*T);
double V = atan2(T,S);
double W;
if(R>0)
{
```

```
        W = -Q/2-sqrt(R);
}
else
{
        W = -Q/2;
}
double X;
if(R>0)
{
        X = 0;
}
else
{
        X = -sqrt(-R);
}
double Y = sqrt(W*W+X*X);
double Z = atan2(X,W);
double AA;
if(U>0)
{
        AA = pow(U,1/3)*cos(V/3);
}
else
{
        AA = 0;
}
double AB;
if(U>0)
{
        AB = pow(U,1/3)*sin(V/3);
}
else
{
        AB = 0;
}
```

```
double AC = AA*AA+AB*AB;
double AD = atan2(AB,AA);
double AE;
if(AC>0)
{
       AE = -P*AA/(3*AC);
}
else
{
       AE = pow(Y,1/3)*cos(Z/3);
}
double G = O+AA+AE;
double H = H0/(1+K1*G+K1*K2*G*G);
double HG = K1*H*G;
double HG2 = K2*HG*G;
f = y-D1*HG/G0-2*D2*HG2/G0;
```

where `D1`, `D2`, `K1` and `K2` stand for $\delta_{\Delta HG}$, $\delta_{\Delta HG2}$, K_1 and K_2, respectively, and running the codes on OriginPro affords the association constants (K_1 and K_2), the chemical shift ($\delta_{\Delta HG}$ and $\delta_{\Delta HG2}$) and sum of squared residuals ($SS_{1:1}$ and $SS_{1:2}$).

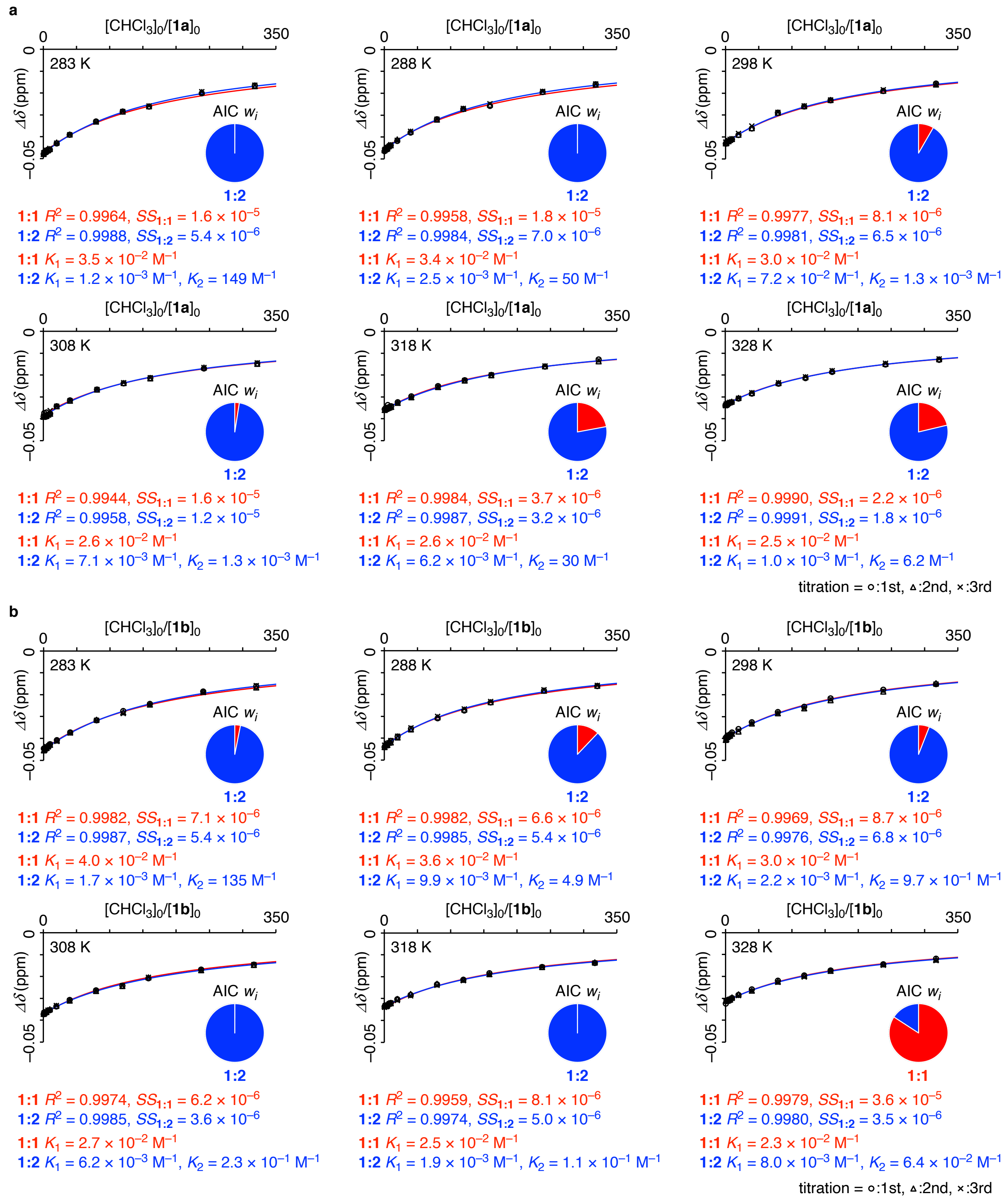

Supplementary Fig. 3 | Variable-temperature titration and fitting analyses. a Data for **1a** and chloroform. **b** Data for **1b** and chloroform.

○Interpretations of spectra from $\delta_{\Delta HG}$ values

By using the $\delta_{\Delta HGn}$ values from the fitting analyses, observed ^{1}H resonances were respectively interpreted for 1:1 and 1:2 models, which showed consistent resonances for the 1:1 models and large

deviations for the 1:2 models. As the present equilibrium is in a fast-exchange regime, a ^{1}H NMR resonance of chloroform during the titration experiments (δ_{avg}) is a time-averaged, weighted resonance of unbound chloroform ($\delta_{unbound}$) and chloroform bound in **1** (δ_{bound}). For **1:1**, because the final value of $\delta_{\Delta HG}$ from the fitting corresponds to "$\delta_{bound} - \delta_{unbound}$", we can derive the fitted chemical shift ($\delta_{avg,fit}$) by using experimental value of 7.11590 ppm for the $\delta_{unbound}$ value and populations of unbound chloroform and bound chloroform from the K_1 value. For **1:2**, because the final values of $\delta_{\Delta HG}$ from the fitting corresponds to "$\delta_{bound,1} - \delta_{unbound}$" and the final values of $\delta_{\Delta HG2}$ from the fitting corresponds to "$\delta_{bound,2} - \delta_{unbound}$", we can derive the fitted chemical shift ($\delta_{avg,fit}$) by using experimental value of 7.11590 ppm for the $\delta_{unbound}$ value and populations of unbound chloroform and bound chloroform (1 and 2) from the K_1 and K_2 values. In Supplementary Figs. 4 and 5, spectra at 298K were shown with the fitted chemical shifts as a representative example. Although the fitted chemical shifts ($\delta_{avg,fit}$; green) from **1:1** and **1:2** reasonably reproduced observed resonances ($\delta_{avg,obs}$) both for **1a** and **1b**, the δ_{bound} values of **1:2** for **1a** and **1b** considerably deviated from each other.

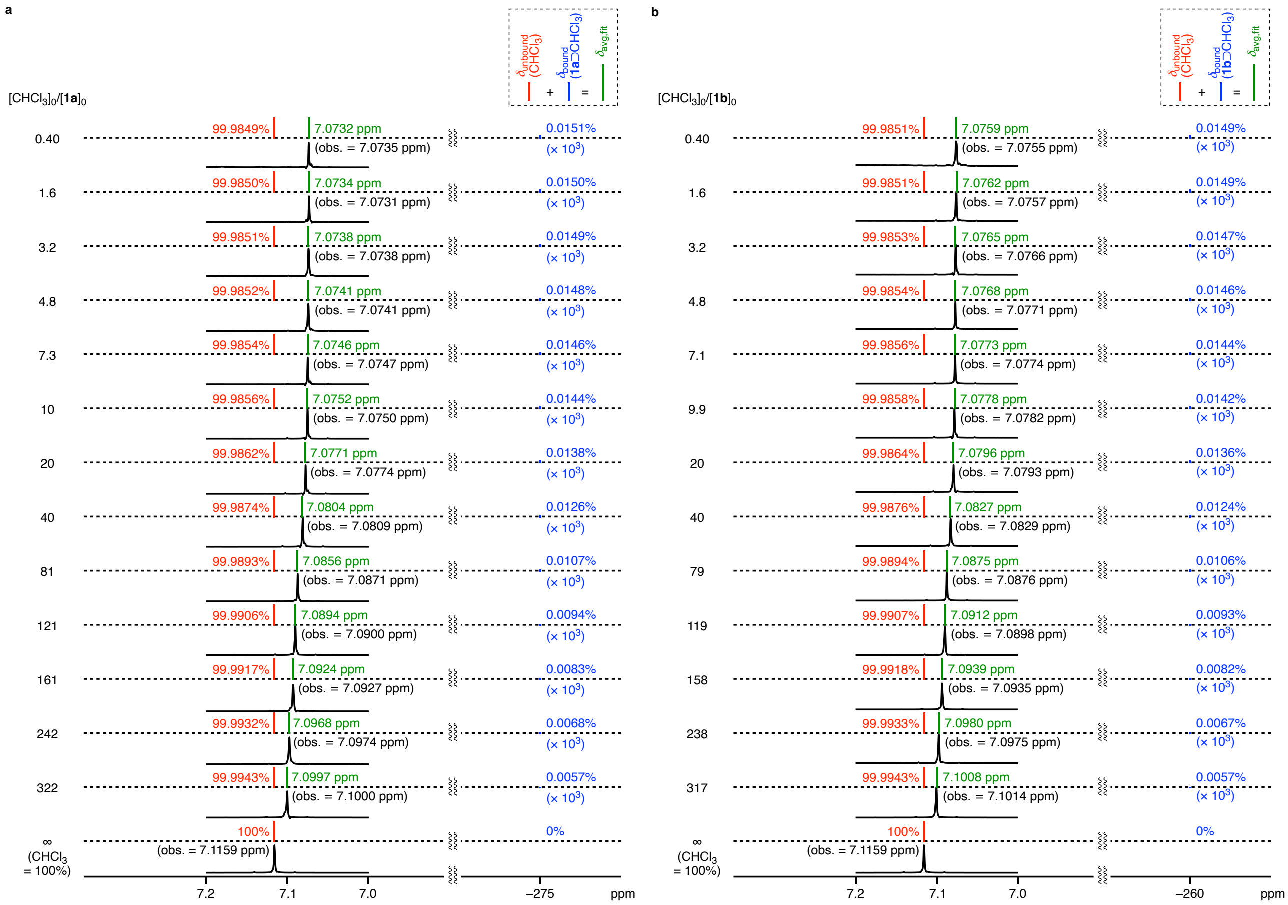

Supplementary Fig. 4 | Chemical shifts of chloroform in 1 ⊃ (CHCl$_3$) complex (1:1). A representative data recorded at 298 K. Experimental spectra are shown with fitted chemical shifts. The height of the bar indicates the population of each chemical species, and the bar for the minor species is magnified by 10^3 times.

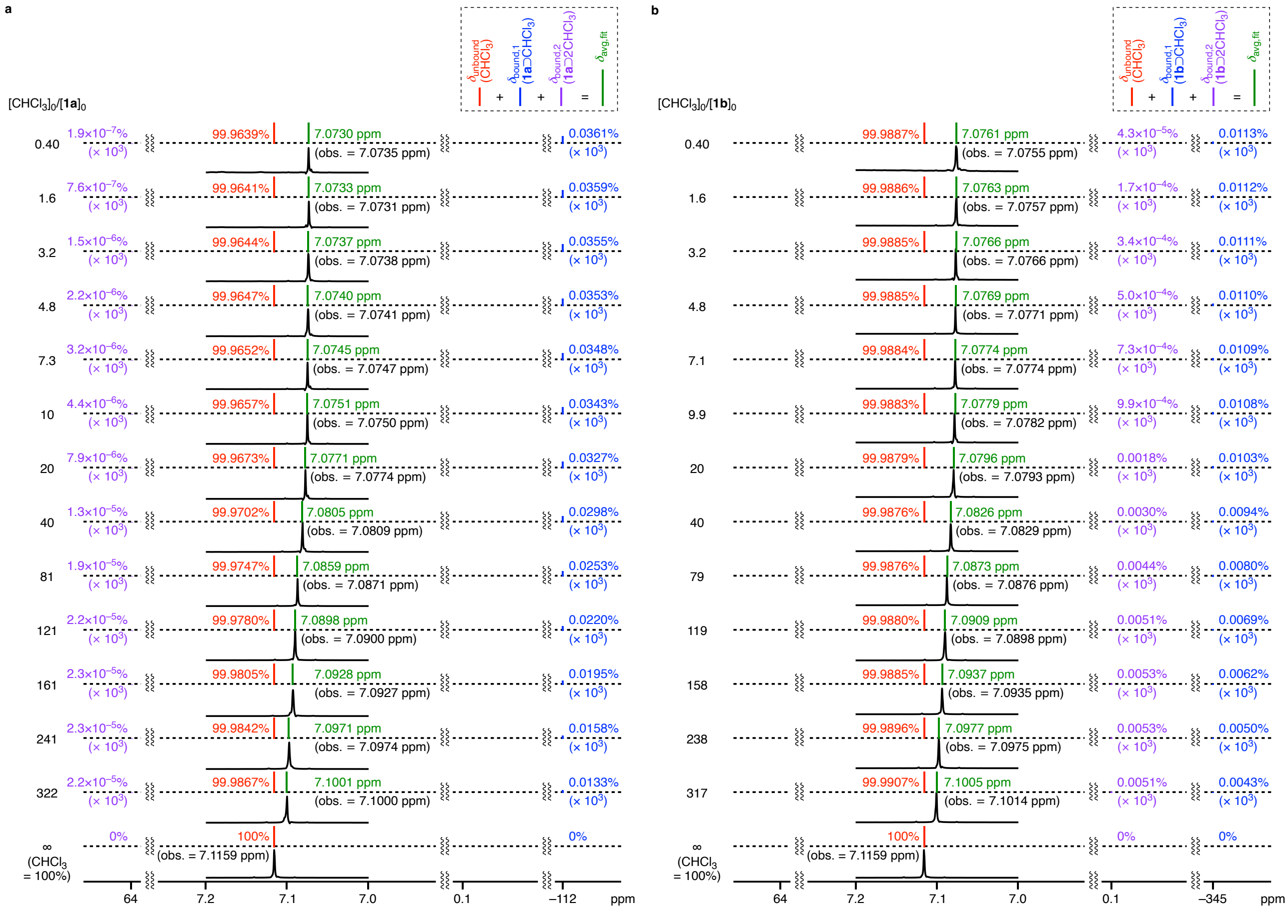

Supplementary Fig. 5 | Chemical shifts of chloroform in 1⊃(CHCl$_3$)$_2$ complex (1:2). A representative data recorded at 298 K. Experimental spectra are shown with fitted chemical shifts. The height of the bar indicates the population of each chemical species, and the bar for the minor species is magnified by 10^3 times.

When we summarized the fitted values ($\delta_{\Delta\mathrm{HG}}$ for **1:1** and $\delta_{\Delta\mathrm{HG}}$ & $\delta_{\Delta\mathrm{HG2}}$ for **1:2**) in a table (Supplementary Table 2), the problems of 1:2 model fitting as well as the credibility of 1:1 fitting became clearer. Details of studies with **1c** are described in the next section, and fitting data are altogether discussed here. The $\delta_{\Delta\mathrm{HG}}$ values for the 1:1 models were consistent for **1a**-**1c** throughout the experimental temperature range (283-328 K) and were obtained from –225 ppm to –296 ppm. The minor deviations were represented by small standard deviation (SD) values associated with the averaged $\delta_{\Delta\mathrm{HG}}$ values. On the other hand, the $\delta_{\Delta\mathrm{HG}}$ and $\delta_{\Delta\mathrm{HG2}}$ values for the 1:2 models considerably varied. For example, the $\delta_{\Delta\mathrm{HG}}$ values for **1a** ranged from –8001 ppm to –110 ppm. The $\delta_{\Delta\mathrm{HG2}}$ values varied from downfield to upfield shifts even for one molecule: a large deviation from –4513 ppm to +0.80 ppm was suggested for **1c**. Although we do not understand the origin of the large $|\delta_{\Delta\mathrm{HG}}|$ values for the 1:1 model, these values were experimentally meaningful within a framework of the present titration experiments. Additional data of relative standard deviations in comparison with re-analyses of $\mathbf{5}_n\supset(C_{60})_m$ further reinforced the conclusion (see below). Importantly, the large $|\delta_{\Delta\mathrm{HG}}|$ values were

essential to determine the small K_1 values. As (8) suggests, for instance, it is the large $|\delta_{\Delta HG}|$ value that makes a small physical change of $\Delta\delta$ from a minute amount of the complex detectable/observable during the titration. The anomalous large values provide an interesting subject to be investigated in the future.

Supplementary Table 2 | The $\delta_{\Delta HGn}$ values from the fitting analyses: 1⊃(CHCl$_3$)$_n$*

	1:1 model	1:2 model
283 K	**1a**: $\delta_{\Delta HG}$ = –271 ppm<br>**1b**: $\delta_{\Delta HG}$ = –225 ppm<br>**1c**: $\delta_{\Delta HG}$ = –255 ppm	**1a**: $\delta_{\Delta HG}$ = –8001 ppm, $\delta_{\Delta HG2}$ = +0.96 ppm<br>**1b**: $\delta_{\Delta HG}$ = –5165 ppm, $\delta_{\Delta HG2}$ = –0.66 ppm<br>**1c**: $\delta_{\Delta HG}$ = –1474 ppm, $\delta_{\Delta HG2}$ = –116 ppm
288 K	**1a**: $\delta_{\Delta HG}$ = –270 ppm<br>**1b**: $\delta_{\Delta HG}$ = –241 ppm<br>**1c**: $\delta_{\Delta HG}$ = –245 ppm	**1a**: $\delta_{\Delta HG}$ = –3614 ppm, $\delta_{\Delta HG2}$ = +2.2 ppm<br>**1b**: $\delta_{\Delta HG}$ = –878 ppm, $\delta_{\Delta HG2}$ = +4.5 ppm<br>**1c**: $\delta_{\Delta HG}$ = –774 ppm, $\delta_{\Delta HG2}$ = –382 ppm
298 K	**1a**: $\delta_{\Delta HG}$ = –282 ppm<br>**1b**: $\delta_{\Delta HG}$ = –267 ppm<br>**1c**: $\delta_{\Delta HG}$ = –266 ppm	**1a**: $\delta_{\Delta HG}$ = –119 ppm, $\delta_{\Delta HG2}$ = +56 ppm<br>**1b**: $\delta_{\Delta HG}$ = –353 ppm, $\delta_{\Delta HG2}$ = –7.0 ppm<br>**1c**: $\delta_{\Delta HG}$ = –3024 ppm, $\delta_{\Delta HG2}$ = –20 ppm
308 K	**1a**: $\delta_{\Delta HG}$ = –296 ppm<br>**1b**: $\delta_{\Delta HG}$ = –277 ppm<br>**1c**: $\delta_{\Delta HG}$ = –272 ppm	**1a**: $\delta_{\Delta HG}$ = –110 ppm, $\delta_{\Delta HG2}$ = –26 ppm<br>**1b**: $\delta_{\Delta HG}$ = –1184 ppm, $\delta_{\Delta HG2}$ = –127 ppm<br>**1c**: $\delta_{\Delta HG}$ = –2904 ppm, $\delta_{\Delta HG2}$ = +0.80 ppm
318 K	**1a**: $\delta_{\Delta HG}$ = –276 ppm<br>**1b**: $\delta_{\Delta HG}$ = –273 ppm<br>**1c**: $\delta_{\Delta HG}$ = –270 ppm	**1a**: $\delta_{\Delta HG}$ = –1142 ppm, $\delta_{\Delta HG2}$ = –1.8 ppm<br>**1b**: $\delta_{\Delta HG}$ = –3591 ppm, $\delta_{\Delta HG2}$ = –562 ppm<br>**1c**: $\delta_{\Delta HG}$ = –787 ppm, $\delta_{\Delta HG2}$ = –3908 ppm
328 K	**1a**: $\delta_{\Delta HG}$ = –270 ppm<br>**1b**: $\delta_{\Delta HG}$ = –275 ppm<br>**1c**: $\delta_{\Delta HG}$ = –275 ppm	**1a**: $\delta_{\Delta HG}$ = –655 ppm, $\delta_{\Delta HG2}$ = –0.84 ppm<br>**1b**: $\delta_{\Delta HG}$ = –782 ppm, $\delta_{\Delta HG2}$ = –137 ppm<br>**1c**: $\delta_{\Delta HG}$ = –1034 ppm, $\delta_{\Delta HG2}$ = –4513 ppm
avg	**1a**: $\delta_{\Delta HG}$ = –277 ± 9 ppm<br>**1b**: $\delta_{\Delta HG}$ = –260 ± 20 ppm<br>**1c**: $\delta_{\Delta HG}$ = –264 ± 11 ppm	**1a**: $\delta_{\Delta HG}$ = –2273 ± 2823 ppm, $\delta_{\Delta HG2}$ = +5.3 ± 24.8 ppm<br>**1b**: $\delta_{\Delta HG}$ = –1992 ± 1764 ppm, $\delta_{\Delta HG2}$ = –138 ± 198 ppm<br>**1c**: $\delta_{\Delta HG}$ = –1666 ± 947 ppm, $\delta_{\Delta HG2}$ = –1490 ± 1936ppm

*The $\delta_{\Delta HGn}$ value at each temperature was obtained from 39 spectra, and the average $\delta_{\Delta HGn}$ value for each molecule was obtained from 234 spectra.

○Evaluations of fitting

Quality of fitting was first analyzed by coefficients of determination (R^2) for the GOF measure by using a following equation,

$$R^2 = 1 - \frac{SS}{\sum_i (\Delta\delta_i - \Delta\delta_{\mathrm{avg}})^2} \quad (17),$$

where $\Delta\delta_i$ is actual $\Delta\delta$ data for a datapoint *i* and $\Delta\delta_{\mathrm{avg}}$ is a total average.

Model credibility was then compared between **1:1** and **1:2** by using $SS_{\mathbf{1:1}}$ and $SS_{\mathbf{1:2}}$.

For the *F*-test, the *F* value was obtained by using the following equation,

$$F = \frac{(SS_{\mathbf{1:1}} - SS_{\mathbf{1:2}})/SS_{\mathbf{1:2}}}{(df_1 - df_2)/df_2} \quad (18),$$

where df_1 and df_2 are the degrees of freedom of **1:1** and **1:2**, respectively. For data with *N* datapoints with *k* parameters, $df = N - k$, and in the present case, *N* is 39, *k* for **1:1** is 2 and *k* for **1:2** is 4. The *P* value was then obtained by the FDIST function of Microsoft Excel with `FDIST(F, df1, df2)`.

For the AIC, the AIC value was obtained by using the following equation,

AIC was calculated by using an equation,

$$\mathrm{AIC} = N \ln\left(\frac{SS}{N}\right) + 2k \quad (19).$$

The w_i value was then obtained by using the following equation,

$$w_i = \frac{\exp\left(\frac{(\mathrm{AIC}_{\mathrm{min}} - \mathrm{AIC}_i)}{2}\right)}{\sum_k \exp\left(\frac{(\mathrm{AIC}_{\mathrm{min}} - \mathrm{AIC}_k)}{2}\right)} \quad (20),$$

where $\mathrm{AIC}_{\mathrm{min}}$ is the smaller value of AIC (**1:2** in the present case), and AIC_i is the AIC value of **1:1** or **1:2**.

○Confirmation experiments with additional congener (**1c**)

Taking account of reviewer comments, the applicability of the present method to another congener, **1c**, was examined. The titration experiments, fitting analyses and van 't Hoff validations were performed as described for **1a**/**1b**, and the results were summarized in Supplementary Figs. 6-9. A 1:1 model for **1c** was similarly supported by the van 't Hoff validaiton, and the fitted $\delta_{\Delta\mathrm{HG}}$ value were also similar to those derived for **1a** and **1b**.

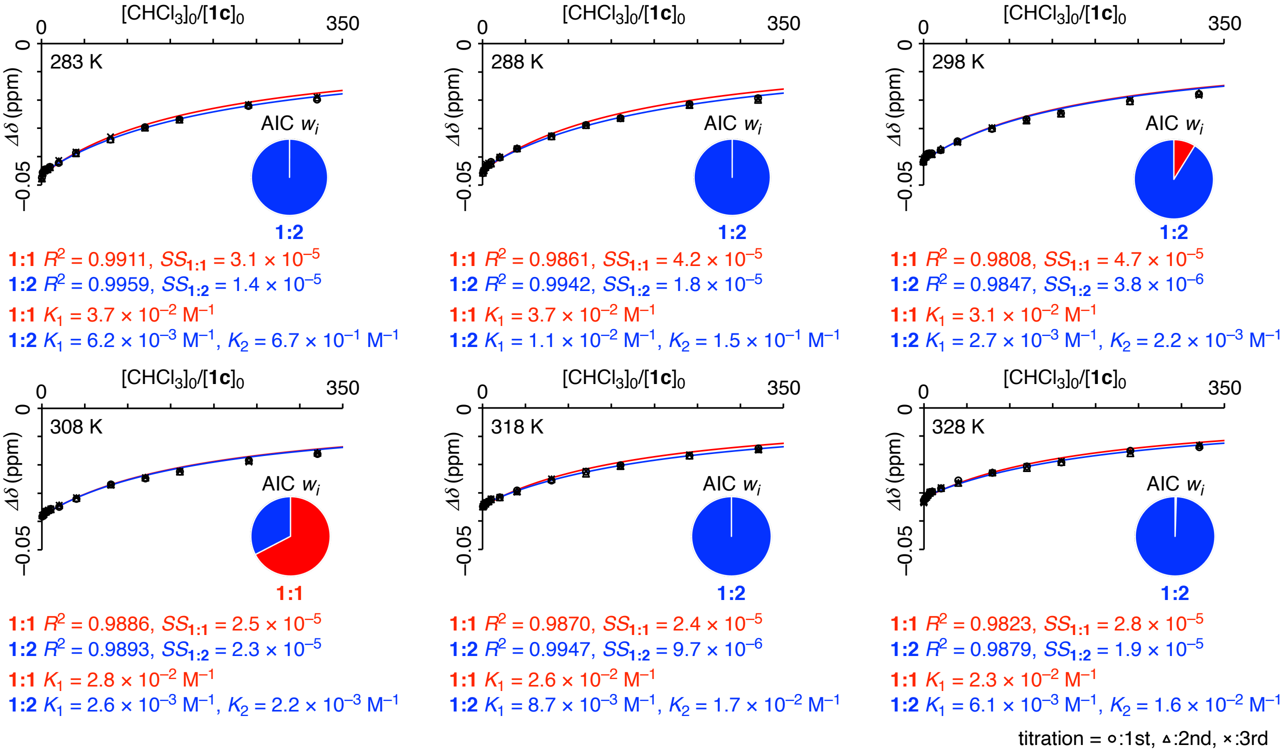

Supplementary Fig. 6 | Variable-temperature titration and fitting analyses. Data for **1c** and chloroform.

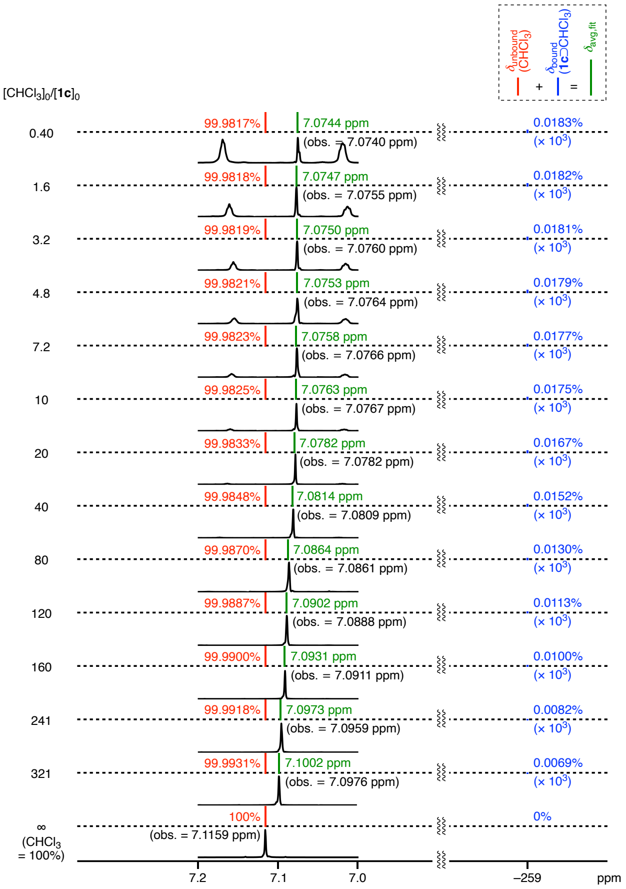

Supplementary Fig. 7 | Chemical shifts of chloroform in 1c ⊃ ($CHCl_3$) complex (1:1). A representative data recorded at 298 K. Experimental spectra are shown with fitted chemical shifts. The height of the bar indicates the population of each chemical species, and the bar for the minor species is magnified by 10^3 times.

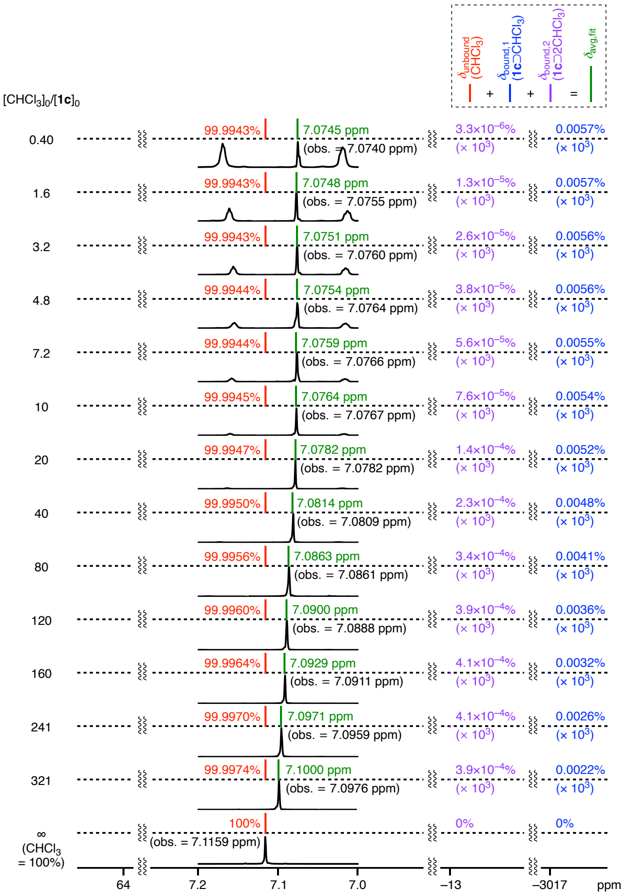

Supplementary Fig. 8 | Chemical shifts of chloroform in 1c ⊃ $(CHCl_3)_2$ complex (1:2). A representative data recorded at 298 K. Experimental spectra are shown with fitted chemical shifts. The height of the bar indicates the population of each chemical species, and the bar for the minor species is magnified by 10^3 times.

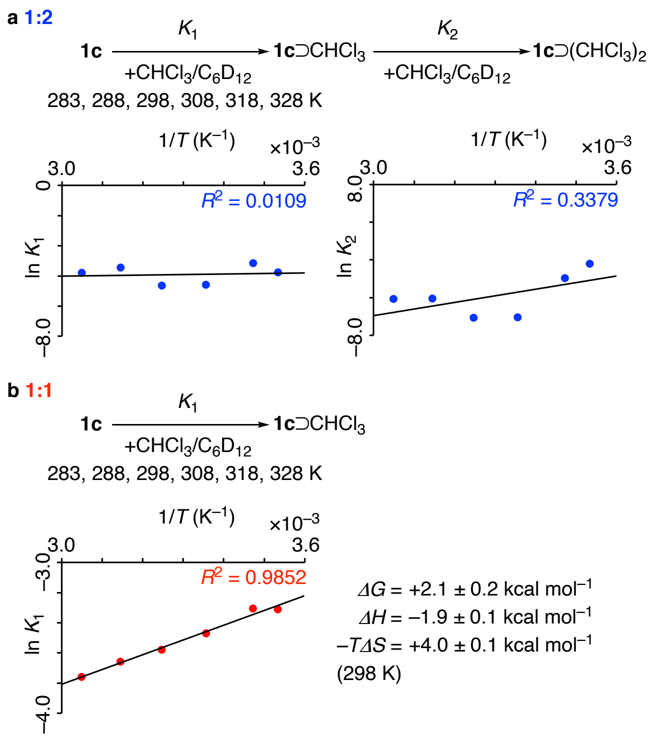

Supplementary Fig. 9 | van 't Hoff validation of variable-temperature titration data for the 1c ⊃(CHCl$_3$)$_n$ complexes. a Validation of the 1:2 stoichiometry. **b** Validation of the 1:1 stoichiometry. Thermodynamic parameters from the van 't Hoff fits are shown.

$\mathbf{5}_n$⊃(C_{60})$_m$

Procedures of re-analyses of reported titration data for $\mathbf{5}_n$⊃(C_{60})$_m$ are described.[3] There were 252 ^{1}H NMR spectra from 3 sets of titration experiments from 14 different ratios of **5** (host) and C_{60} (guest) under 6 different temperature conditions. Total concentrations of host and guest during the titration are defined as $[H]_0$ and $[G]_0$ (observables), respectively. Experiments were performed under the continuous variation method conditions with $[H]_0 + [G]_0 = 1 \times 10^{-4}$ M, and $[H]_0$ and $[G]_0$ are thus described in equations as

$$[\mathrm{H}]_0 = \frac{10^{-4}}{1+x} \quad (21),$$

$$[\mathrm{G}]_0 = [\mathrm{H}]_0 x \quad (22),$$

where $x = [G]_0/[H]_0$.

○1:1 complex, **5**⊃(C_{60})$_1$

During the titration, we experimentally track the change of the chemical shift of host ($\Delta\delta$), which obeys an equation

$$\Delta\delta = \delta_{\Delta \mathrm{HG}} \frac{[\mathrm{HG}]}{[\mathrm{H}]_0} \quad (23).$$

where $\delta_{\Delta HG}$ is a chemical shift of 1:1 complex. By applying (7) to (23), we obtain

$$\Delta\delta = \frac{\delta_{\Delta HG}}{2}\left\{\left(1+\frac{[G]_0}{[H]_0}+\frac{1}{K_1[H]_0}\right)-\sqrt{\left(1+\frac{[G]_0}{[H]_0}+\frac{1}{K_1[H]_0}\right)^2-\frac{4[G]_0}{[H]_0}}\right\} \quad (24),$$

which is used for the fitting analyses.

Plotting the observed $\Delta\delta$ values on the y axis against $[G]_0/[H]_0$ in the x axis affords isotherms in the $[C_{60}]_0/[\mathbf{5}]_0$-$\Delta\delta$ graphs (Supplementary Fig. 11), which were fitted by using (24). Specifically, using (21), (22) and (24), the codes to minimize "`f`" are written as follows:

```
double H0 = 0.0001/(x+1);
double G0 = H0*x;
f=y-0.5*D*((1+G0/H0+1/(K*H0))-sqrt((1+G0/H0+1/(K*H0))^2-4*G0/H0));
```

where `D` stands for $\delta_{\Delta HG}$ and `K` stands for K_1, and running the codes on OriginPro affords the association constant (K_1), the chemical shift ($\delta_{\Delta HG}$) and sum of squared residuals ($SS_{1:1}$).

○1:2 complex, $\mathbf{5}\supset(C_{60})_2$

During the titration, we experimentally track the change of the chemical shift of host ($\Delta\delta$), which obeys an equation

$$\Delta\delta = \delta_{\Delta HG}\frac{[HG]}{[H]_0} + \delta_{\Delta HG_2}\frac{[HG_2]}{[H]_0} \quad (25),$$

where $\delta_{\Delta HG}$ is a difference of chemical shift of host and 1:1 complex, and $\delta_{\Delta HG2}$ is a difference of chemical shift of host and 1:2 complex. By applying (10)-(13) to (25), we obtain

$$\Delta\delta = \frac{\delta_{\Delta HG}K_1[G] + \delta_{\Delta HG_2}K_1K_2[G]^2}{1+K_1[G]+K_1K_2[G]^2} \quad (26),$$

which is used for the fitting analyses.

Plotting the observed $\Delta\delta$ values on the y axis against $[G]_0/[H]_0$ in the x axis affords isotherms in the $[C_{60}]_0/[\mathbf{5}]_0$-$\Delta\delta$ graphs (Supplementary Fig. 11), which were fitted by using (26). Specifically, using (10)-(14), (21), (22), (25) and (26), the codes to minimize "`f`" are written as follows:

```
double H0 = 0.0001/(x+1);
double G0 = H0*x;
// Start of coefficient of equation
double K = K1*K2;
double L = K1+K1*K2*(2*H0-G0);
double M = K1*(H0-G0)+1;
double N = -G0;
// End of coefficient of equation
```

```
double O = -L/(3*K);
double P = M/K-L*L/(3*K*K);
double Q = N/K-M*L/(3*K*K)+2*L*L*L/(27*K*K*K);
double R = Q*Q/4+P*P*P/27;
double S;
if(R>0)
{
        S = -Q/2+sqrt(R);
}
        else
{
        S = -Q/2;
}
double T;
if(R>0)
{
        T = 0;
}
else
{
        T = sqrt(-R);
}
double U = sqrt(S*S+T*T);
double V = atan2(T,S);
double W;
if(R>0)
{
        W = -Q/2-sqrt(R);
}
else
{
        W = -Q/2;
}
double X;
if(R>0)
```

```
{
       X = 0;
}
else
{
       X = -sqrt(-R);
}
double Y = sqrt(W*W+X*X);
double Z = atan2(X,W);
double AA;
if(U>0)
{
       AA = pow(U,1/3)*cos(V/3);
}
else
{
       AA = 0;
}
double AB;
if(U>0)
{
       AB = pow(U,1/3)*sin(V/3);
}
else
{
       AB = 0;
}
double AC = AA*AA+AB*AB;
double AD = atan2(AB,AA);
double AE;
if(AC>0)
{
       AE = -P*AA/(3*AC);
}
else
```

```
{
        AE = pow(Y,1/3)*cos(Z/3);
}
double G = O+AA+AE;
double H = H0/(1+K1*G+K1*K2*G*G);
double HG = K1*H*G;
double HG2 = K2*HG*G;
f = y-D1*HG/H0-D2*HG2/H0;
```

where D1, D2, K1 and K2 stand for $\delta_{\Delta HG}$, $\delta_{\Delta HG2}$, K_1 and K_2, respectively, and running the codes on OriginPro affords the association constants (K_1 and K_2), the chemical shift ($\delta_{\Delta HG}$ and $\delta_{\Delta HG2}$) and sum of squared residuals ($SS_{1:1}$ and $SS_{1:2}$).

○2:1 complex, (**5**)$_2$⊃C_{60}

Upon formation of 2:1 complex, the total concentration of host is defined as a sum of concentrations of host ([H]), 1:1 complex ([HG]) and 2:1 complex ([H_2G]) in solution, and the total concentration of guest is defined as a sum of concentrations of guest ([G]), 1:1 complex ([HG]) and 2:1 complex ([H_2G]) in solution.

$$[\mathrm{H}]_0 = [\mathrm{H}] + [\mathrm{HG}] + 2[\mathrm{H_2G}] \quad (27).$$

$$[\mathrm{G}]_0 = [\mathrm{G}] + [\mathrm{HG}] + [\mathrm{H_2G}] \quad (28).$$

For 2:1 complex, the association constants K_1 and K_2 are then defined as

$$K_1 = \frac{[\mathrm{HG}]}{[\mathrm{H}][\mathrm{G}]} \quad (29),$$

$$K_2 = \frac{[\mathrm{H_2G}]}{[\mathrm{HG}][\mathrm{H}]} \quad (30).$$

By using (27)-(30), the association constant and the total concentrations are correlated in a cubic equation

$$K_1K_2[\mathrm{H}]^3 + (2K_1K_2[\mathrm{G}]_0 - K_1K_2[\mathrm{H}]_0 + K_1)[\mathrm{H}]^2 + (K_1[\mathrm{G}]_0 - K_1[\mathrm{H}]_0 + 1)[\mathrm{H}] - [\mathrm{H}]_0 = 0 \quad (31).$$

During the titration, we experimentally track the change of the chemical shift of host ($\Delta\delta$), which obeys an equation

$$\Delta\delta = \delta_{\Delta\mathrm{HG}}\frac{[\mathrm{HG}]}{[\mathrm{H}]_0} + 2\delta_{\Delta\mathrm{H_2G}}\frac{[\mathrm{H_2G}]}{[\mathrm{H}]_0} \quad (32),$$

where $\delta_{\Delta HG}$ is a difference of chemical shift of host and 1:1 complex, and $\delta_{\Delta H2G}$ is a difference of chemical shift of host and 2:1 complex. By applying (27)-(30) to (32), we obtain

$$\Delta\delta = \frac{\delta_{\Delta HG} K_1 [H] + 2\delta_{\Delta H_2G} K_1 K_2 [H]^2}{1 + K_1 [H] + K_1 K_2 [H]^2} \cdot \frac{[G]_0}{[H]_0} \quad (33),$$

which is used for the fitting analyses.

Plotting the observed $\Delta\delta$ values on the *y* axis against $[G]_0/[H]_0$ in the *x* axis affords isotherms in the $[C_{60}]_0/[\mathbf{5}]_0$-$\Delta\delta$ graphs (Supplementary Fig. 11), which were fitted by using (33). Specifically, using (21), (22), (27)-(33), the codes to minimize "`f`" are written as follows:

```
double H0 = 0.0001/(x+1);
double G0 = H0*x;
// Start of coefficient of equation
double K = K1*K2;
double L = K1+K1*K2*(2*G0-H0);
double M = K1*(G0-H0)+1;
double N = -H0;
// End of coefficient of equation
double O = -L/(3*K);
double P = M/K-L*L/(3*K*K);
double Q = N/K-M*L/(3*K*K)+2*L*L*L/(27*K*K*K);
double R = Q*Q/4+P*P*P/27;
double S;
if(R>0)
{
      S = -Q/2+sqrt(R);
}
      else
{
      S = -Q/2;
}
double T;
if(R>0)
{
      T = 0;
}
else
{
      T = sqrt(-R);
```

```
}
double U = sqrt(S*S+T*T);
double V = atan2(T,S);
double W;
if(R>0)
{
       W = -Q/2-sqrt(R);
}
else
{
       W = -Q/2;
}
double X;
if(R>0)
{
       X = 0;
}
else
{
       X = -sqrt(-R);
}
double Y = sqrt(W*W+X*X);
double Z = atan2(X,W);
double AA;
if(U>0)
{
       AA = pow(U,1/3)*cos(V/3);
}
else
{
       AA = 0;
}
double AB;
if(U>0)
{
```

```
        AB = pow(U,1/3)*sin(V/3);
}
else
{
        AB = 0;
}
double AC = AA*AA+AB*AB;
double AD = atan2(AB,AA);
double AE;
if(AC>0)
{
        AE = -P*AA/(3*AC);
}
else
{
        AE = pow(Y,1/3)*cos(Z/3);
}
double H = O+AA+AE;
double G = G0/(1+K1*H+K1*K2*H*H);
double HG = K1*H*G;
double H2G = K2*HG*H;
f = y-D1*HG/H0-2*D2*H2G/H0;
```

where `D1`, `D2`, `K1` and `K2` stand for $\delta_{\Delta HG}$, $\delta_{\Delta H2G}$, K_1 and K_2, respectively, and running the codes on OriginPro affords the association constants (K_1 and K_2), the chemical shift ($\delta_{\Delta HG}$ and $\delta_{\Delta H2G}$) and sum of squared residuals ($SS_{1:1}$ and $SS_{2:1}$).

By using the $\delta_{\Delta HG}$ values from the fitting analyses, observed ^{1}H resonances were interpreted for 1:1 (Supplementary Fig. 10).

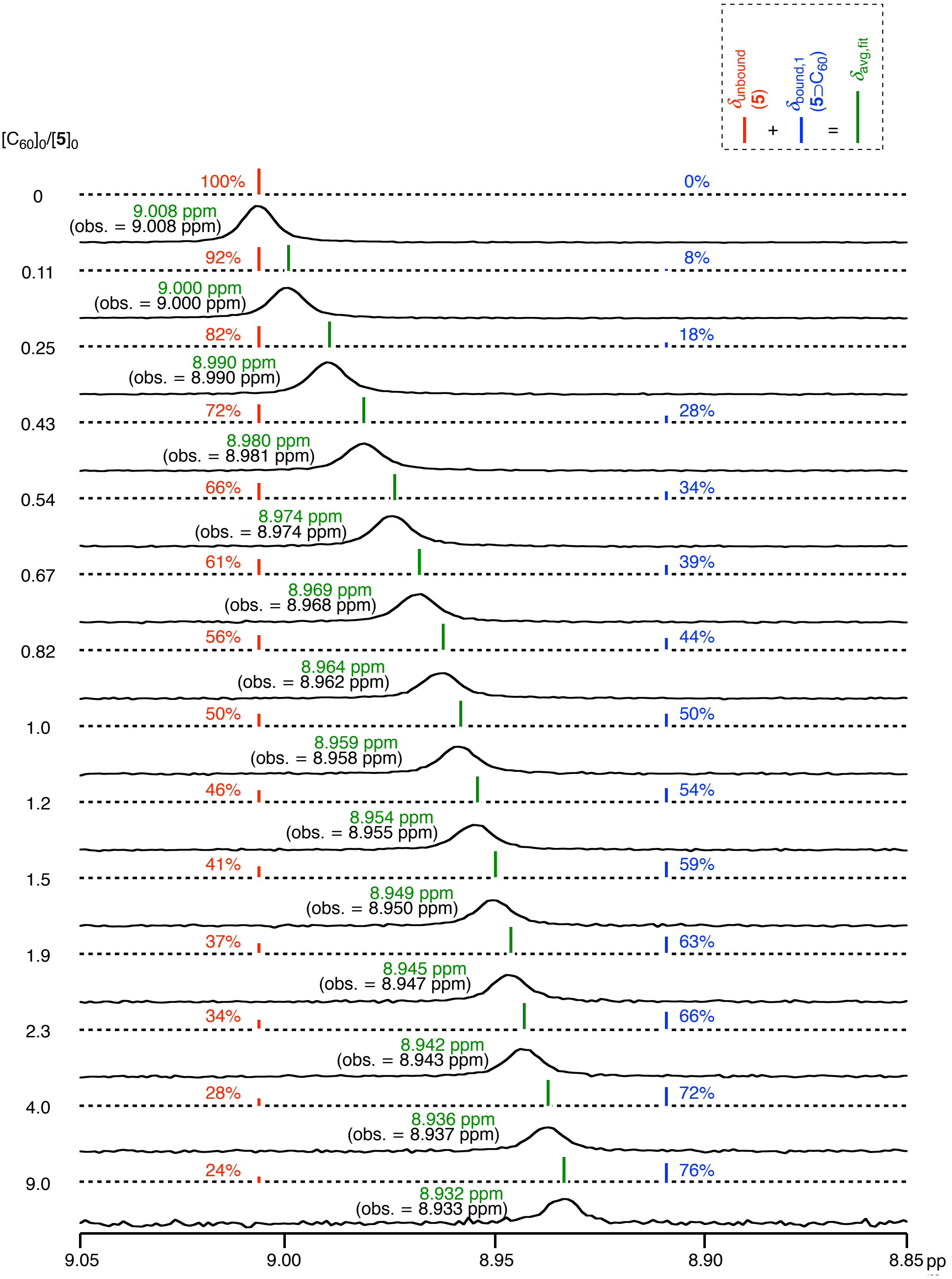

Supplementary Fig. 10| Chemical shifts of 5⊃(C$_{60}$) complex (1:1). A representative data recorded at 298 K. Experimental spectra are shown with fitted chemical shifts. The height of the bar indicates the population of each chemical species.

The fitted $\delta_{\Delta H_nG_m}$ values for $\mathbf{5}_n\supset(C_{60})_m$ were summarized in a table (Supplementary Table 3).

Supplementary Table 3 | The $\delta_{\Delta H_nG_m}$ values from the fitting analyses: $\mathbf{5}_n\supset(C_{60})_m$*

	1:1 model	1:2 model	2:1 model
283 K	$\delta_{\Delta HG}$ = –0.100 ppm	$\delta_{\Delta HG}$ = –0.103 ppm<br>$\delta_{\Delta HG2}$ = –0.0755 ppm	$\delta_{\Delta HG}$ = –0.0990 ppm<br>$\delta_{\Delta H2G}$ = +0.055 ppm
293 K	$\delta_{\Delta HG}$ = –0.102 ppm	$\delta_{\Delta HG}$ = –0.0947 ppm<br>$\delta_{\Delta HG2}$ = –0.0787 ppm	$\delta_{\Delta HG}$ = –0.0989 ppm<br>$\delta_{\Delta H2G}$ = +0.14 ppm
298 K	$\delta_{\Delta HG}$ = –0.0997 ppm	$\delta_{\Delta HG}$ = –0.105 ppm<br>$\delta_{\Delta HG2}$ = –0.0673 ppm	$\delta_{\Delta HG}$ = –0.100 ppm<br>$\delta_{\Delta H2G}$ = –0.10 ppm
303 K	$\delta_{\Delta HG}$ = –0.0995 ppm	$\delta_{\Delta HG}$ = –0.128 ppm<br>$\delta_{\Delta HG2}$ = –0.0643 ppm	$\delta_{\Delta HG}$ = –0.101 ppm<br>$\delta_{\Delta H2G}$ = –0.20 ppm
313 K	$\delta_{\Delta HG}$ = –0.0965 ppm	$\delta_{\Delta HG}$ = –0.108 ppm<br>$\delta_{\Delta HG2}$ = –0.0640 ppm	$\delta_{\Delta HG}$ = –0.0966 ppm<br>$\delta_{\Delta H2G}$ = –0.050 ppm
323 K	$\delta_{\Delta HG}$ = –0.0951 ppm	$\delta_{\Delta HG}$ = –0.0946 ppm<br>$\delta_{\Delta HG2}$ = –0.346 ppm	$\delta_{\Delta HG}$ = –0.0959 ppm<br>$\delta_{\Delta H2G}$ = –0.18 ppm
avg	$\delta_{\Delta HG}$ = –0.099 ± 0.002 ppm	$\delta_{\Delta HG}$ = –0.11 ± 0.01 ppm<br>$\delta_{\Delta HG2}$ = –0.12 ± 0.10 ppm	$\delta_{\Delta HG}$ = –0.099 ± 0.002 ppm<br>$\delta_{\Delta H2G}$ = –0.057 ± 0.124 ppm

*The $\delta_{\Delta H_nG_m}$ value at each temperature was obtained from 42 spectra, and the average $\delta_{\Delta H_nG_m}$ value for each molecule was obtained from 252 spectra.

When we compared $\delta_{\Delta H_nG_m}$ values of "**1** + $CHCl_3$" and "**5** + C_{60}" in detail, we found additional data that supported the credibility of the $\delta_{\Delta HG}$ values for $\mathbf{1}\supset(CHCl_3)_1$. From the average values with standard deviations, we can derive the relative standard deviations (%RSD) that are commonly used as a standardized measure of dispersion. The %RSD values for four systems were calculated for the average values and were shown in Supplementary Table 4. The fitting with a reasonable model (1:1) showed a small dispersion in a range of 2-8%, but the fitting with unreasonable models (1:2/2:1) showed a large dispersion such as 468% (1:2 of **1a** + $CHCl_3$) at the maximum. Thus, these values confirmed the credibility of $\delta_{\Delta HG}$ values for the "**1** + $CHCl_3$" systems.

Supplementary Table 4 | Relative standard deviation of $\delta_{\Delta\mathrm{H}n\mathrm{G}m}$ values*

	**1a** + $CHCl_3$	**1b** + $CHCl_3$	**1c** + $CHCl_3$	**5** + C_{60}
1:1 model	3.2%	7.7%	4.0%	2.0%
1:2 model	124% ($\delta_{\Delta\mathrm{HG}}$)<br>468% ($\delta_{\Delta\mathrm{HG2}}$)	89% ($\delta_{\Delta\mathrm{HG}}$)<br>143% ($\delta_{\Delta\mathrm{HG2}}$)	57% ($\delta_{\Delta\mathrm{HG}}$)<br>130% ($\delta_{\Delta\mathrm{HG2}}$)	9.1% ($\delta_{\Delta\mathrm{HG}}$)<br>83% ($\delta_{\Delta\mathrm{HG2}}$)
2:1 model	-	-	-	2.0% ($\delta_{\Delta\mathrm{HG}}$)<br>218% ($\delta_{\Delta\mathrm{H2G}}$)

*See Supplementary Tables 2 and 3 for the original data.

Quality of fitting and model credibility of the "**5** + C_{60}" system were then analyzed by following procedures described for **1**⊃$(CHCl_3)_n$ (Supplementary Fig. 11).

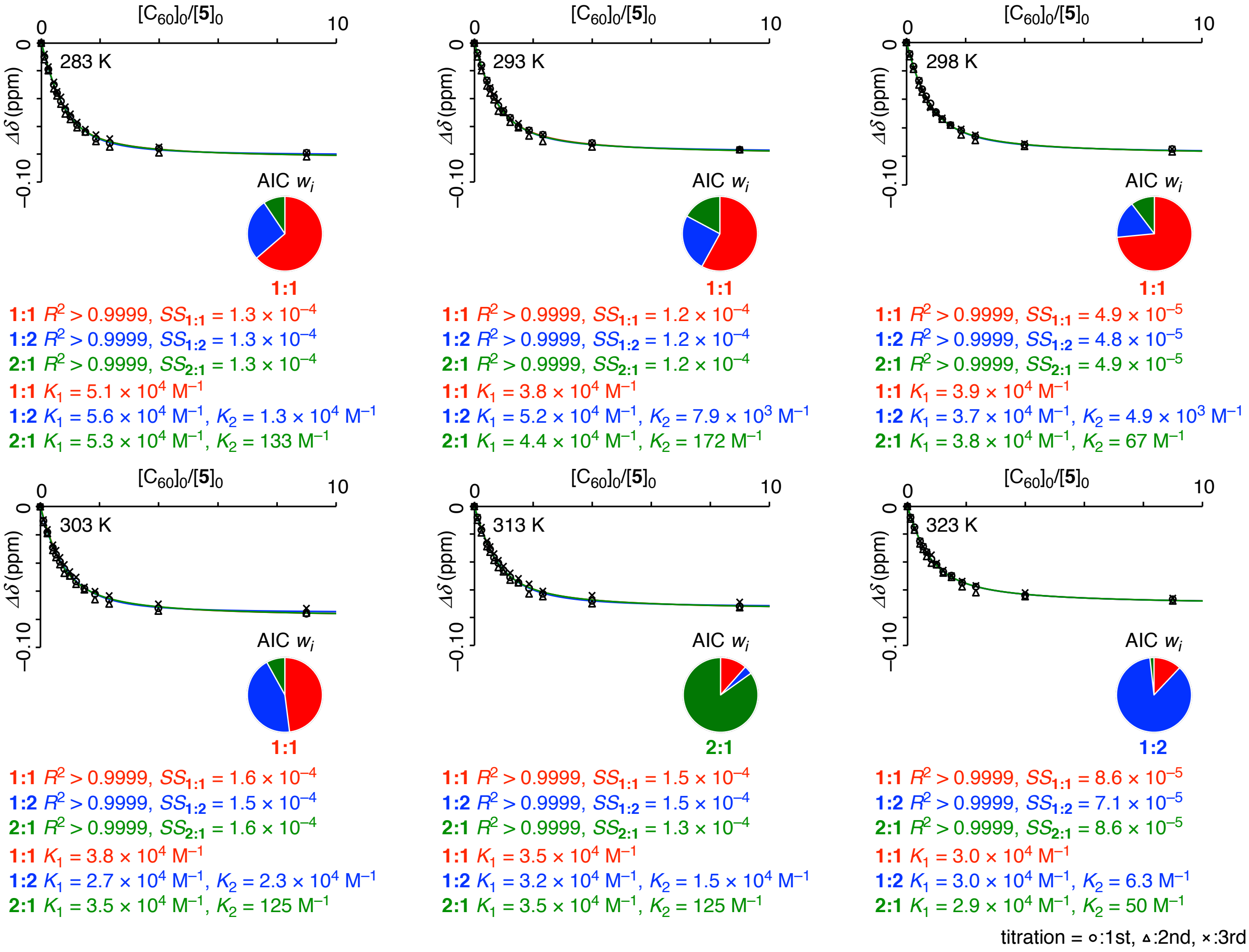

Supplementary Fig. 11 | Variable-temperature titration and fitting analyses. Data for **5** and C_{60}.

For fitting analyses, many other programs are also available. For example, one reviewer recommended a web-based application, BindFit (http://app.supramolecular.org/bindfit/), and a more recent application, SupraFit (https://doi.org/10.1002/cmtd.202200006), was also found.[4] We also provided easy-to-use Excel files with detailed manuals for fitting/AIC analyses in our previous study (https://doi.org/10.1002/anie.202219059).[5] Nonetheless, those who perform fitting analyses should pay close attentions to stages and their background science (Supplementary Table 5).

Supplementary Table 5 | Stages of fitting analyses

stage	scientific ground	method	
1.	isotherm fitting	thermodynamics	$\Delta\delta = f(K_n)$
2.	GOF of isotherm fitting	statistics	R^2
3.	comparison of isotherm fitting	statistics	*F*-test/*P*-value
3.	comparison of isotherm fitting	information-theoretic	AIC/w_i value
4.	K_n values	thermodynamics	from stage 1
5.	van 't Hoff validation	thermodynamics	1/*T*-ln *K* plot

Theoretical calculations

Theoretical structures of **1a**⊃($CHCl_3$) and **1b**⊃($CHCl_3$) are compared with *t*-Bu substituents replaced by Me groups as the model. CH-π hydrogen bonds were also compared via AIM analyses (Supplementary Fig. 12).

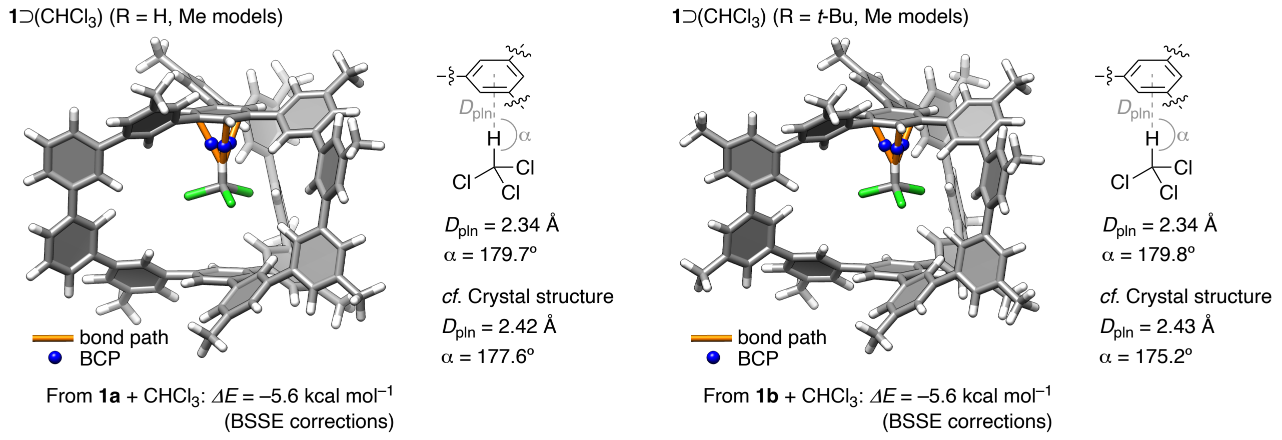

Supplementary Fig. 12 | Theoretical structures of 1⊃($CHCl_3$).

Spectra

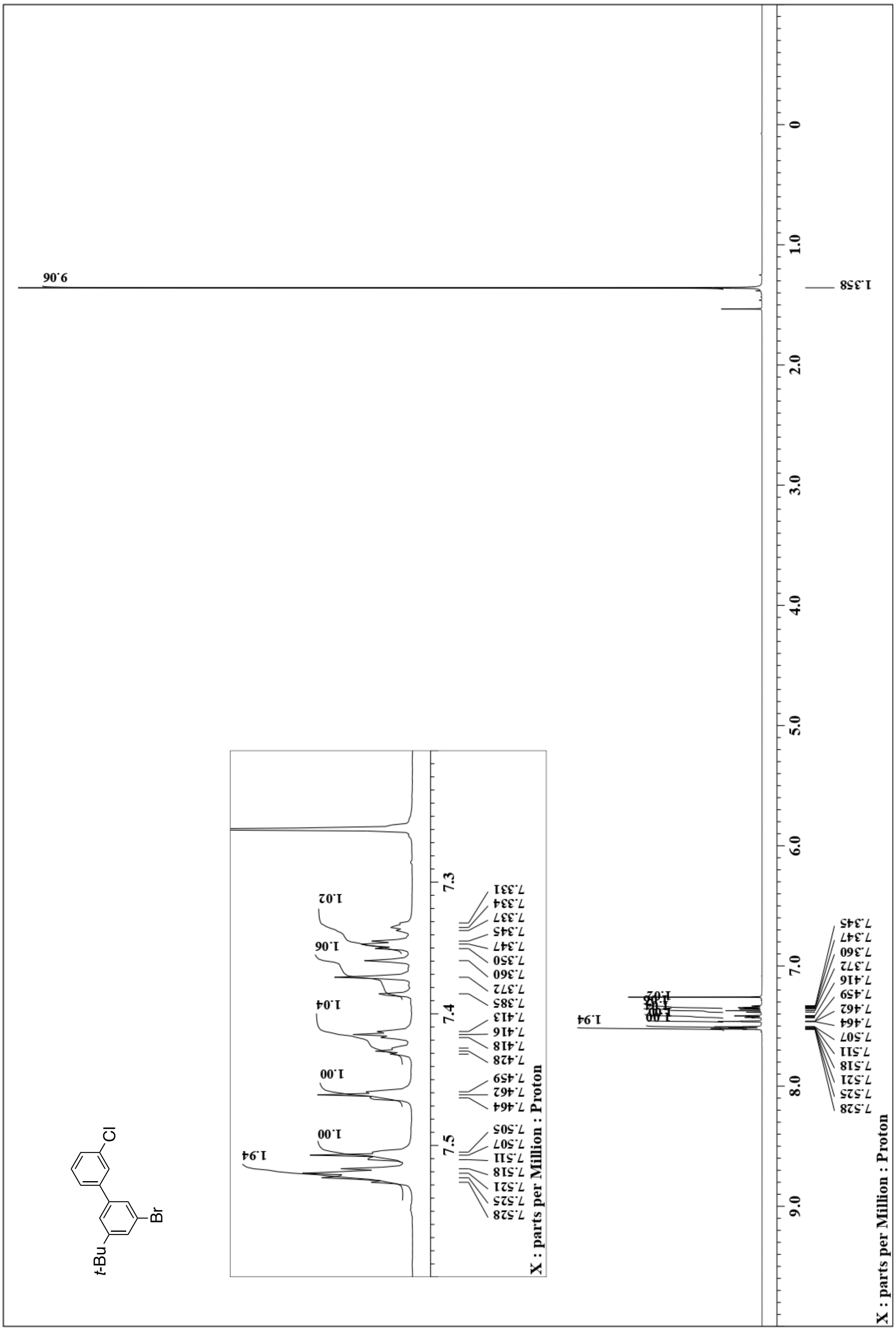

Supplementary Fig. 13 | ^{1}H NMR spectrum of compound 3 ($CDCl_3$, 600 MHz, 298 K).

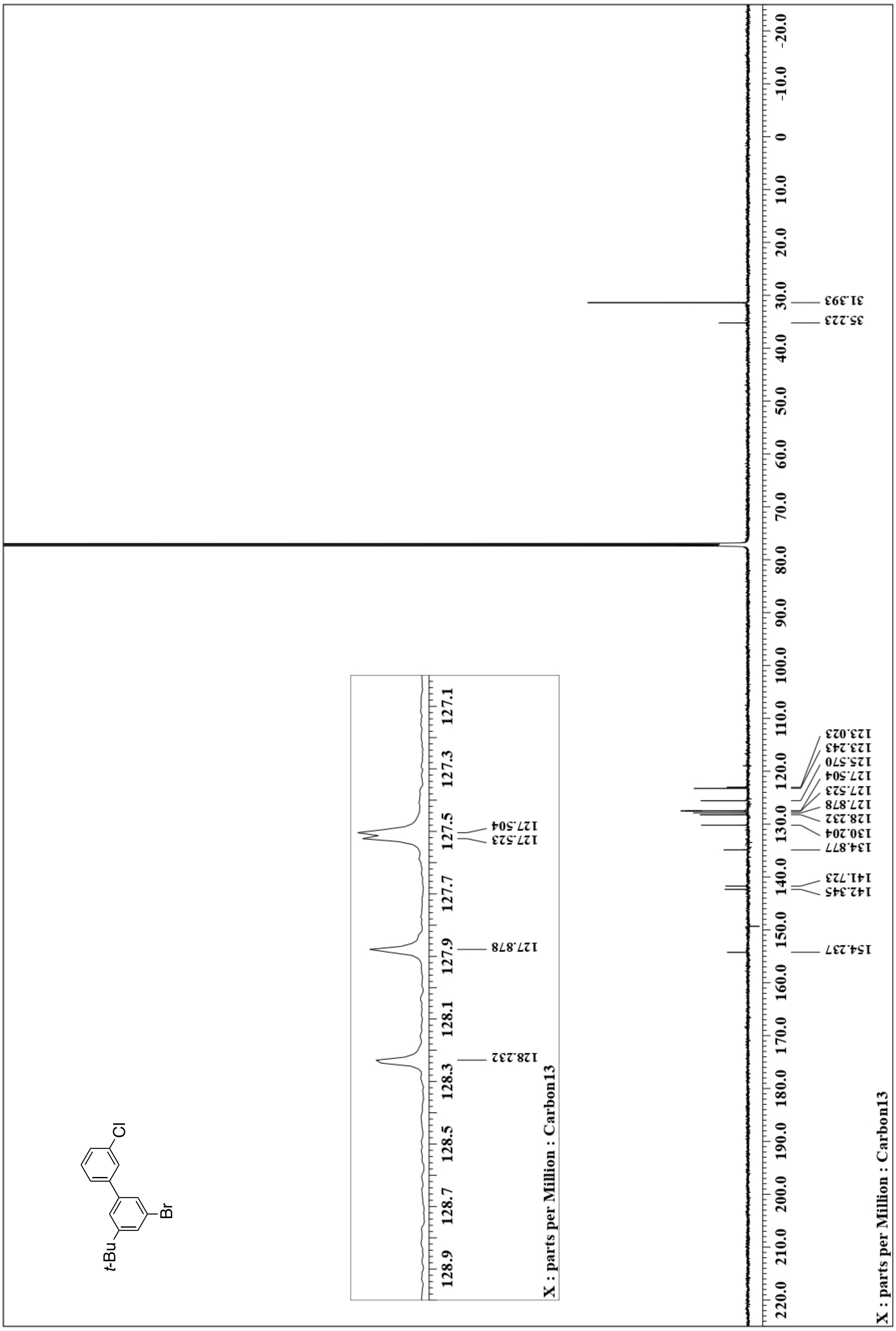

Supplementary Fig. 14 | ^{13}C NMR spectrum of compound 3 ($CDCl_3$, 151 MHz, 298 K).

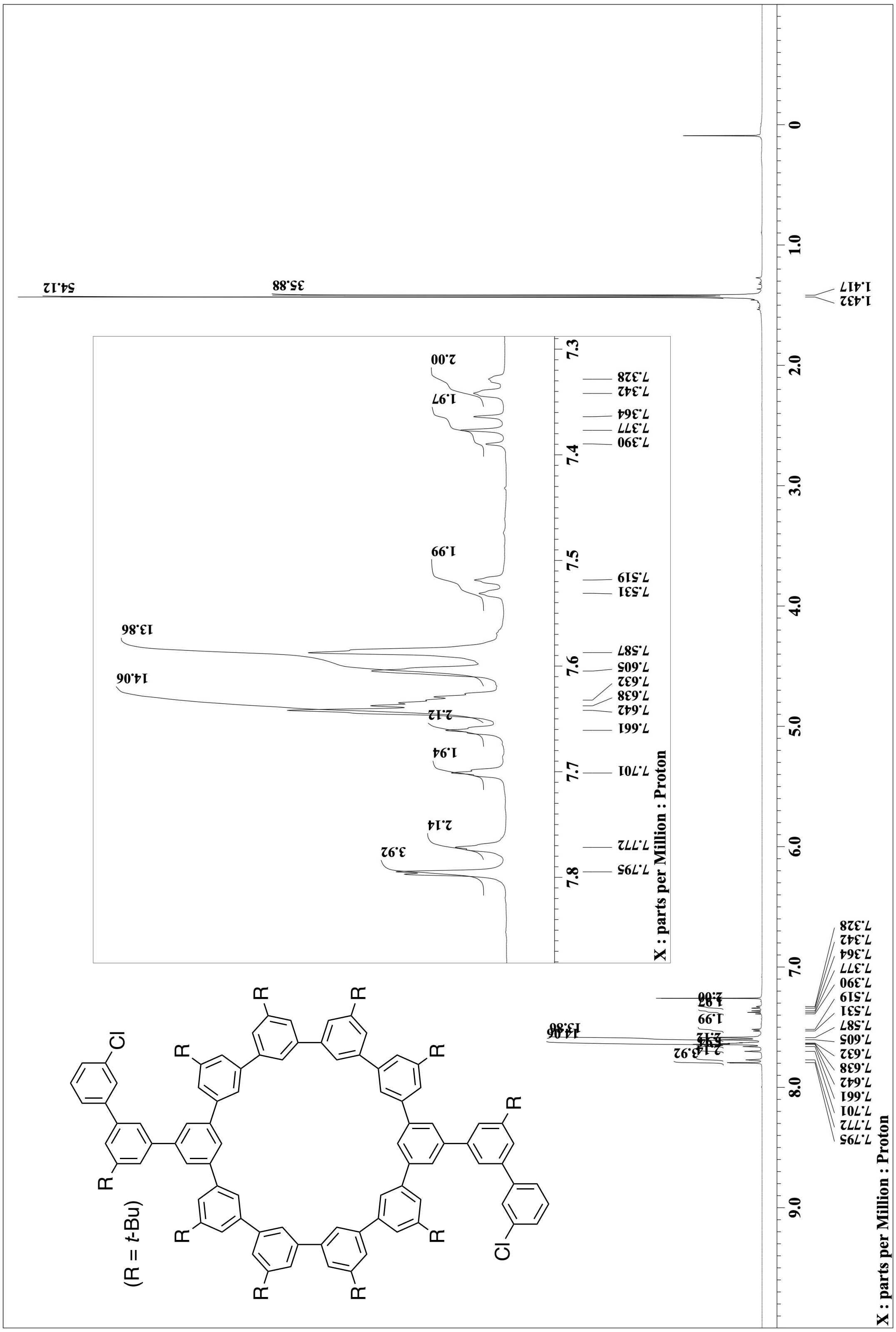

Supplementary Fig. 15 | ^{1}H NMR spectrum of compound 4 ($CDCl_3$, 600 MHz, 298 K).

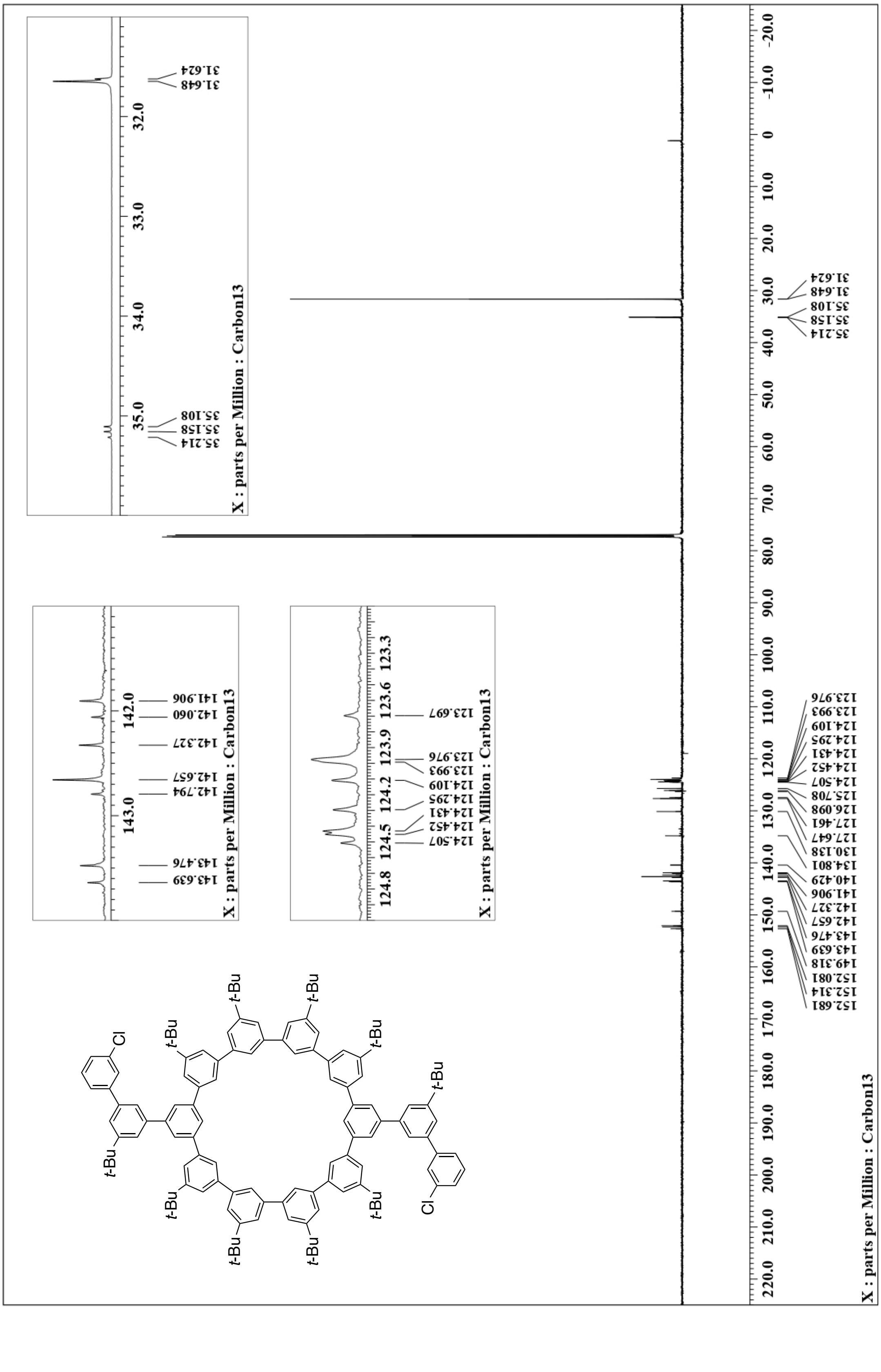

Supplementary Fig. 16 | ^{13}C NMR spectrum of compound 4 ($CDCl_3$, 151 MHz, 298 K).

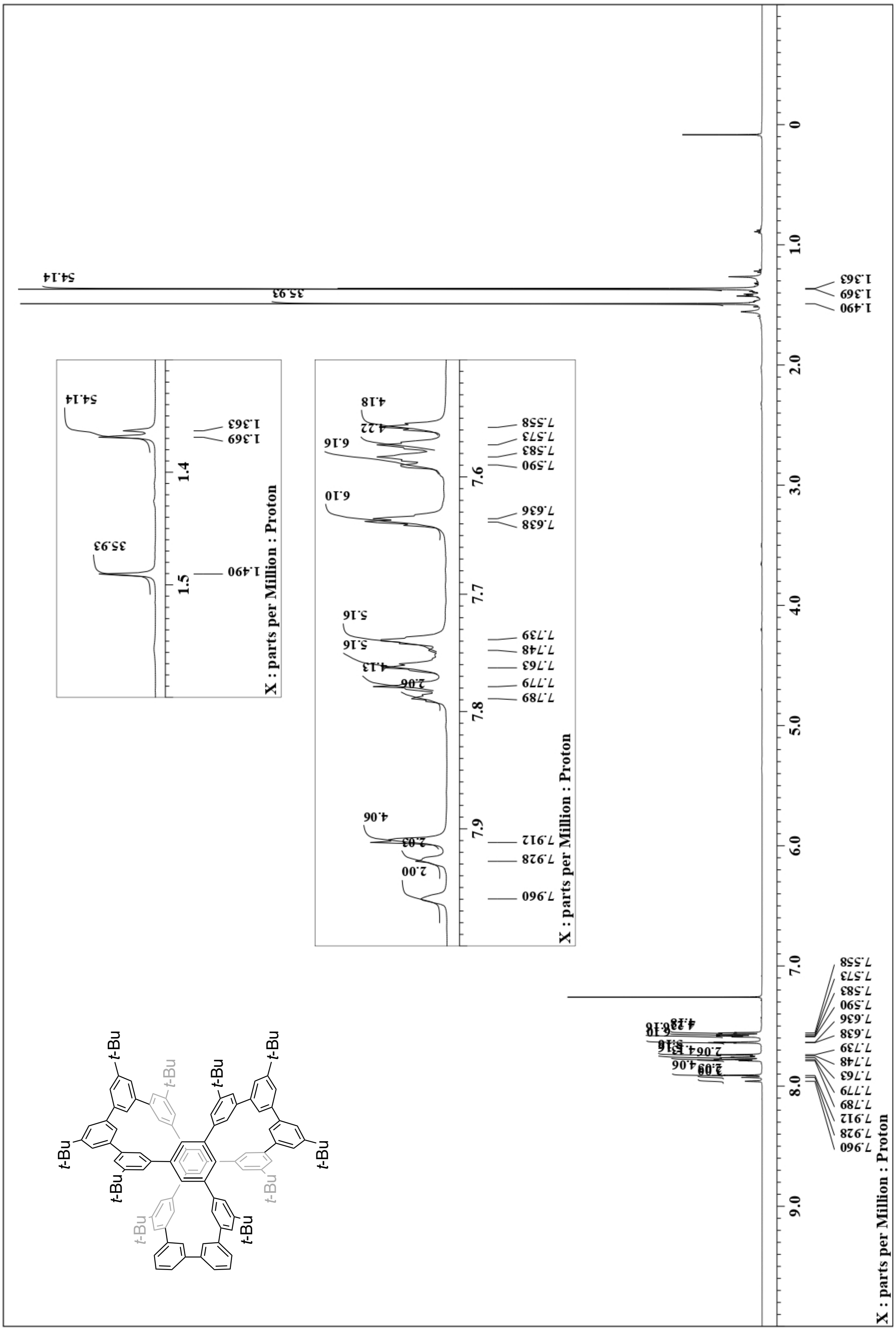

Supplementary Fig. 17 | ¹H NMR spectrum of compound 1a ($CDCl_3$, 600 MHz, 298 K).

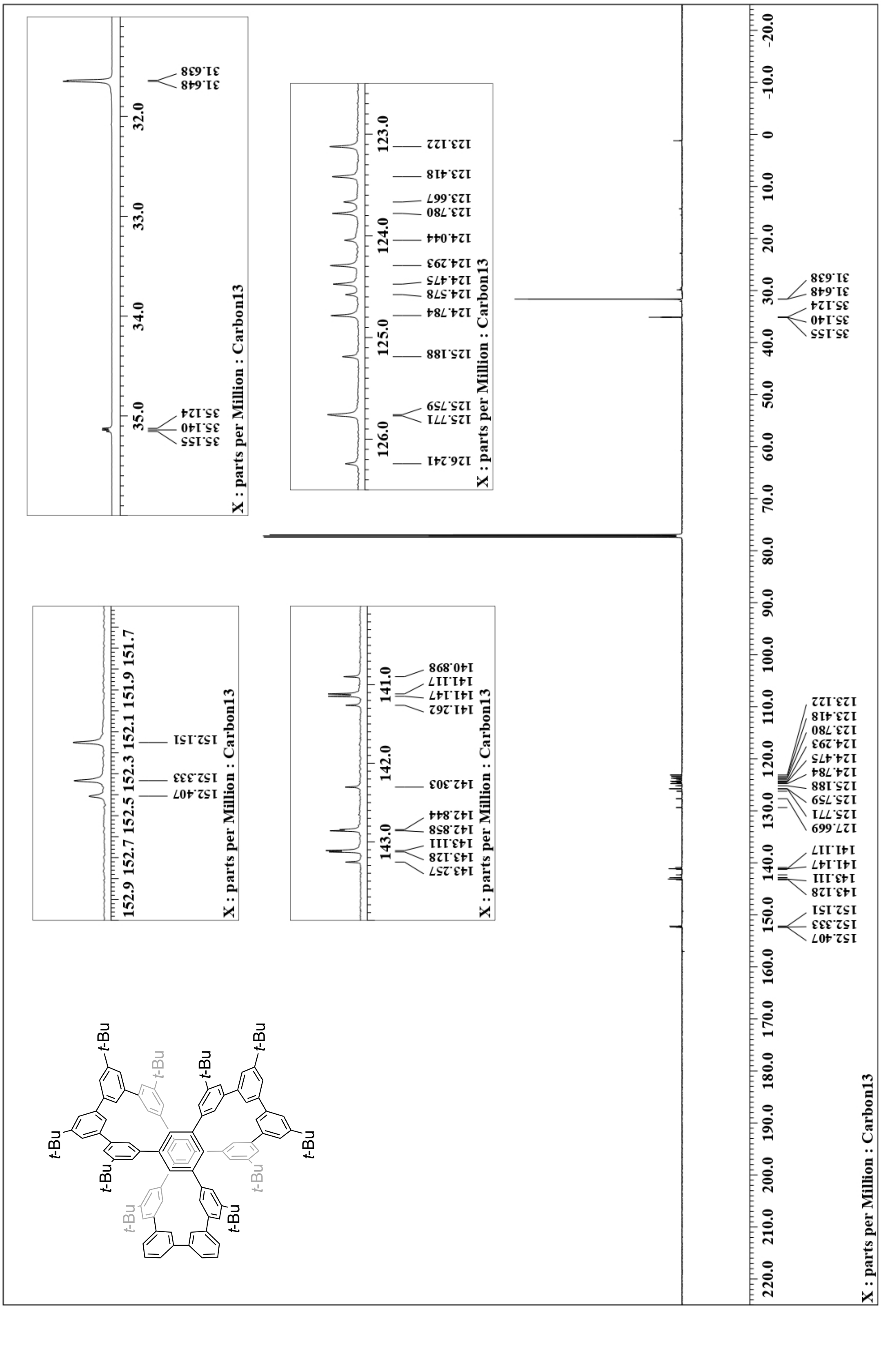

Supplementary Fig. 18 | ^{13}C NMR spectrum of compound 1a ($CDCl_3$, 151 MHz, 298 K).

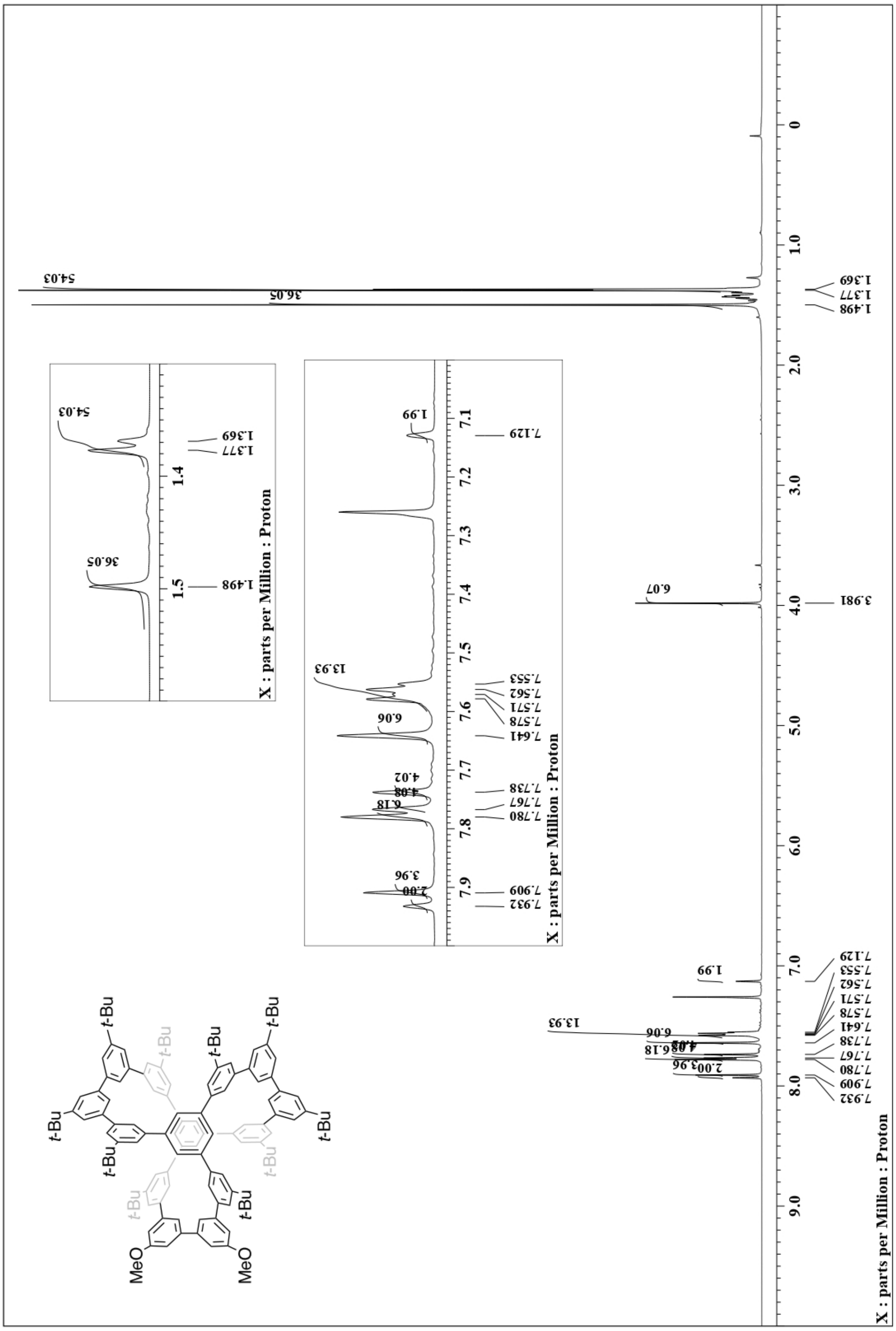

Supplementary Fig. 19 | ^{1}H NMR spectrum of compound 1c ($CDCl_3$, 600 MHz, 298 K).

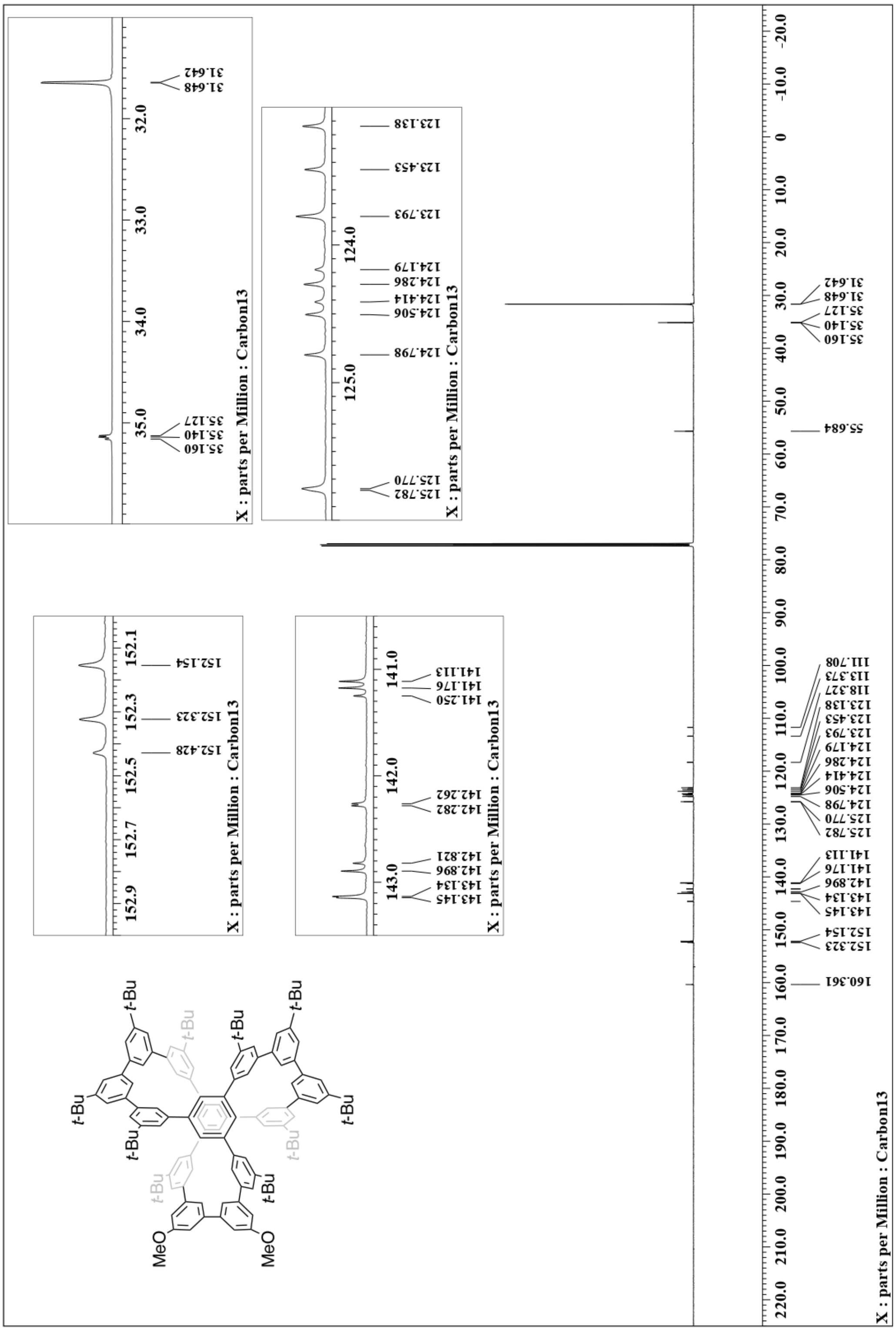

Supplementary Fig. 20 | ^{13}C NMR spectrum of compound 1c ($CDCl_3$, 600 MHz, 298 K).

Supplementary References

1 Pangborn, A. B., Giardello, M. A., Grubbs, R. H., Rosen, R. K. & Timmers, F. J. Safe and convenient procedure for solvent purification. *Organometallics* **15**, 1518-1520 (1996).

2 Fukunaga, T. M., Kato, T., Ikemoto, K. & Isobe, H. A minimal cage of a diamond twin with chirality. *Proc. Natl. Acad. Sci. USA* **119**, e2120160119 (2022).

3 Ikemoto, K., Kobayashi, R., Sato, S. & Isobe, H. Entropy-driven ball-in-bowl assembly of fullerene and geodesic phenylene bowl. *Org. Lett.* **19**, 2362-2365 (2017).

4 Hübler, C. Analysing binding stoichiometries in NMR titration experiments using Monte Carlo simulation and resampling techniques. *PeerJ Anal. Chem.* **4**, e23 (2022).

5 Ikemoto, K., Takahashi, K., Ozawa, T. & Isobe, H. Akaike's information criterion for stoichiometry inference of supramolecular complexes. *Angew. Chem. Int. Ed.* **62**, e202219059 (2023).
